# Delayed microglial depletion after spinal cord injury reduces chronic inflammation and neurodegeneration in the brain and improves neurological recovery in male mice

**DOI:** 10.7150/thno.49199

**Published:** 2020-09-14

**Authors:** Yun Li, Rodney M. Ritzel, Niaz Khan, Tuoxin Cao, Junyun He, Zhuofan Lei, Jessica J. Matyas, Boris Sabirzhanov, Simon Liu, Hui Li, Bogdan A. Stoica, David J. Loane, Alan I. Faden, Junfang Wu

**Affiliations:** 1 Department of Anesthesiology and Center for Shock, Trauma and Anesthesiology Research (STAR), University of Maryland School of Medicine, Baltimore, MD, 21201 USA.; 2 University of Maryland Center to Advance Chronic Pain Research, University of Maryland, Baltimore, MD, 21201 USA.

**Keywords:** spinal cord injury, microglia, CSF1R, cognition, depression

## Abstract

Neuropsychological deficits, including impairments in learning and memory, occur after spinal cord injury (SCI). In experimental SCI models, we and others have reported that such changes reflect sustained microglia activation in the brain that is associated with progressive neurodegeneration. In the present study, we examined the effect of pharmacological depletion of microglia on posttraumatic cognition, depressive-like behavior, and brain pathology after SCI in mice.

**Methods:** Young adult male C57BL/6 mice were subjected to moderate/severe thoracic spinal cord contusion. Microglial depletion was induced with the colony-stimulating factor 1 receptor (CSF1R) antagonist PLX5622 administered starting either 3 weeks before injury or one day post-injury and continuing through 6 weeks after SCI. Neuroinflammation in the injured spinal cord and brain was assessed using flow cytometry and NanoString technology. Neurological function was evaluated using a battery of neurobehavioral tests including motor function, cognition, and depression. Lesion volume and neuronal counts were quantified by unbiased stereology.

**Results:** Flow cytometry analysis demonstrated that PLX5622 pre-treatment significantly reduced the number of microglia, as well as infiltrating monocytes and neutrophils, and decreased reactive oxygen species production in these cells from injured spinal cord at 2-days post-injury. Post-injury PLX5622 treatment reduced both CD45^int^ microglia and CD45^hi^ myeloid counts at 7-days. Following six weeks of PLX5622 treatment, there were substantial changes in the spinal cord and brain transcriptomes, including those involved in neuroinflammation. These alterations were associated with improved neuronal survival in the brain and neurological recovery.

**Conclusion:** These findings indicate that pharmacological microglia-deletion reduces neuroinflammation in the injured spinal cord and brain, improving recovery of cognition, depressive-like behavior, and motor function.

## Introduction

Traumatic spinal cord injury (SCI) leads to long-term motor dysfunction, neuropathic pain and/or autonomic nervous system abnormalities 1-5. In addition, clinical evidence indicates substantial cognitive impairment, including deficits in memory span, executive functioning, attention, processing speed, and learning ability, as well as stimulus detection and evaluation 6-9. Recent large cohort studies show that cognitive impairment after SCI is 13-fold greater than controls 10, with a significantly greater risk of subsequent dementia 7. Moreover, SCI commonly leads to mood disorders, including anxiety and depression 11, 12. The high prevalence of cognitive impairment and depression in SCI population is increasingly recognized as serious secondary complications that can impede not only quality of life but also recovery. Recent experimental models of SCI 13-17 show cognitive impairment and depressive-like behavior that parallel those observed clinically. Remarkably, despite this extensive evidence, neither clinical nor experimental studies to date have addressed these changes at a mechanistic level with a view toward limiting such deficits.

Injuries to the spinal cord trigger signaling cascades that result in microglial and astrocyte activation, neuronal cell death, and neuroinflammation, among others 18; these delayed and often-progressive secondary injury processes contribute to tissue loss and neurological dysfunction 19-21. Clinical and experimental studies further show that widespread profound alterations to the brain occur after SCI, which may contribute to the development of cognitive and mood disorders. We and others have previously reported 14-17, 22-25 that chronic inflammation after SCI occurs not only in pain-related pathways such as thalamus, but also in the regions not directly connected to the injury site - such as pre-frontal cortex and hippocampus - that are critical for the processing of emotional, as well as learning and memory-related information. Recently, reducing posttraumatic inflammation has been shown to improve mood in patients with SCI 26. However, the precise molecular mechanisms underlying these changes have not been elucidated.

Neuroinflammation at the injury site reflects both reactive resident microglia and infiltrating monocytes from the blood. Following injury microglia can play either a restorative or destructive role, depending upon injury severity and the relative balance between expressed neuroprotective versus neurotoxic factors 27, 28. More chronically, it is now recognized that dysregulated microglial activation may continue for months to years after CNS trauma 27-34, and can be associated with chronic neurodegeneration. SCI can cause chronic microglial activation in various brain regions 14, 22-25; such neurotoxic inflammation is associated with neuronal endoplasmic reticulum (ER) stress 15, contributing to neurodegeneration and neurological dysfunction.

Microglia are dependent on colony-stimulating factor 1 receptor (CSF1R) signaling for survival in the healthy adult central nervous system 35, 36. Recently identified CSF1R antagonists lead to the rapid and continued elimination of microglia from the CNS 37. Mice lacking microglia, by administration of CSF1R small molecule inhibitors appear healthy and phenotypically normal. Microglial depletion, using this approach, appears beneficial in various CNS disease models 38-42, indicating that CSF1R antagonists may be effective therapeutically for various CNS disorders associated with neuroinflammation. Assessment of the effects of microglial elimination in SCI models have mainly focused on the injury site, with differential treatment paradigms, CSF1R inhibitors, and experimental injury models. Therefore, the resulting conclusions are ranging from those reporting detrimental effects to those showing protection 43, 44. Infiltrating cells at the injury site can contribute to SCI pathology; blocking these cells improves locomotor function 45-49. Systemic application of CSF1R inhibitor PLX5622 can alter circulating monocytes 50, 51 and causes transient blood neutropenia after SCI 43. However, whether PLX5622 affects infiltration of peripheral cells in injury site after SCI is not well studied. Moreover, whether systemic depletion of microglia affects SCI-mediated brain neuroinflammation and/or post-injury cognitive and depressive-like impairments is unknown.

In the present study, we used either pre- or post-injury PLX5622 treatment paradigms in a well-characterized mouse impact SCI model to examine its effects on infiltrating monocytes, brain inflammation, and post-injury cognitive and depressive-like behavior. We demonstrate that PLX5622 treatment reduces not only the number of microglia but also infiltrating monocytes and neutrophils during first week after SCI. Production of reactive oxygen species (ROS) in these cells is significantly reduced with treatment. Moreover, long-term depletion of microglia improves neuronal survival in the brain and neurological recovery; these changes are associated with altered gene pathways involved in neuroinflammation. These findings strongly implicate chronic neurotoxic inflammation as a major pathophysiological factor in SCI-mediated brain pathology and neurological impairments, and support a potential therapeutic role for posttraumatic microglial depletion.

## Methods

### SCI model and drug treatment

All surgical and experimental procedures were performed under protocols approved by the Institutional Animal Care and Use Committee (IACUC) at the University of Maryland, School of Medicine. The majority of experimental SCI studies use females because of the reduced risk of bladder infection 52, 53. However, human SCI is more common in men (~80%) according to the 2019 SCI Data Sheet from the National SCI Statistical Center. Thus, male mice were included in the present study. All experiments were conducted using young adult male C57BL/6 mice (10-12 weeks, Jackson Laboratories), or Cx3xr1-GFP male mice (B6.129P-Cx3cr1tm1Litt/J, Cat# 005582, Jackson Laboratories). Cx3xr1-GFP knock-in/knock-out mice express EGFP in monocytes, dendritic cells, NK cells, and brain microglia under control of the endogenous Cx3cr1 locus. Mice were housed on a 12:12 h light/dark cycle with food and water freely available ad libitum. Moderate/severe spinal cord contusion injury was conducted using the Infinite Horizon (Precision Systems and Instrumentation) spinal cord impactor as previously described 15, 16. Briefly, mice were anesthetized with isoflurane and the spinal column was stabilized using bilateral steel clamps over the lateral processes at T9 and T11. A laminectomy was performed followed by a midline spinal contusion at T10 level with a force of 60 or 70 kdyn, moderate/severe injury. Sham animals underwent the same procedure as SCI mice except for the laminectomy and impact. The bladders of injured mice were manually voided 2-3 times daily for the duration of experiments. Body weight and food weight were monitored daily (short term experiments) or weekly (long-term experiments). After SCI, all mice were assigned to a treatment group according to a randomized block experimental design. Individuals performed functional assessment and involved in data analysis were blinded to group designations throughout all stages of the experiment. The number of mice at various time points in each study is indicated in the figure legends. To minimize stress and fatigue, each animal was subjected to only one behavioral test on a given day.

**Figure 1:**
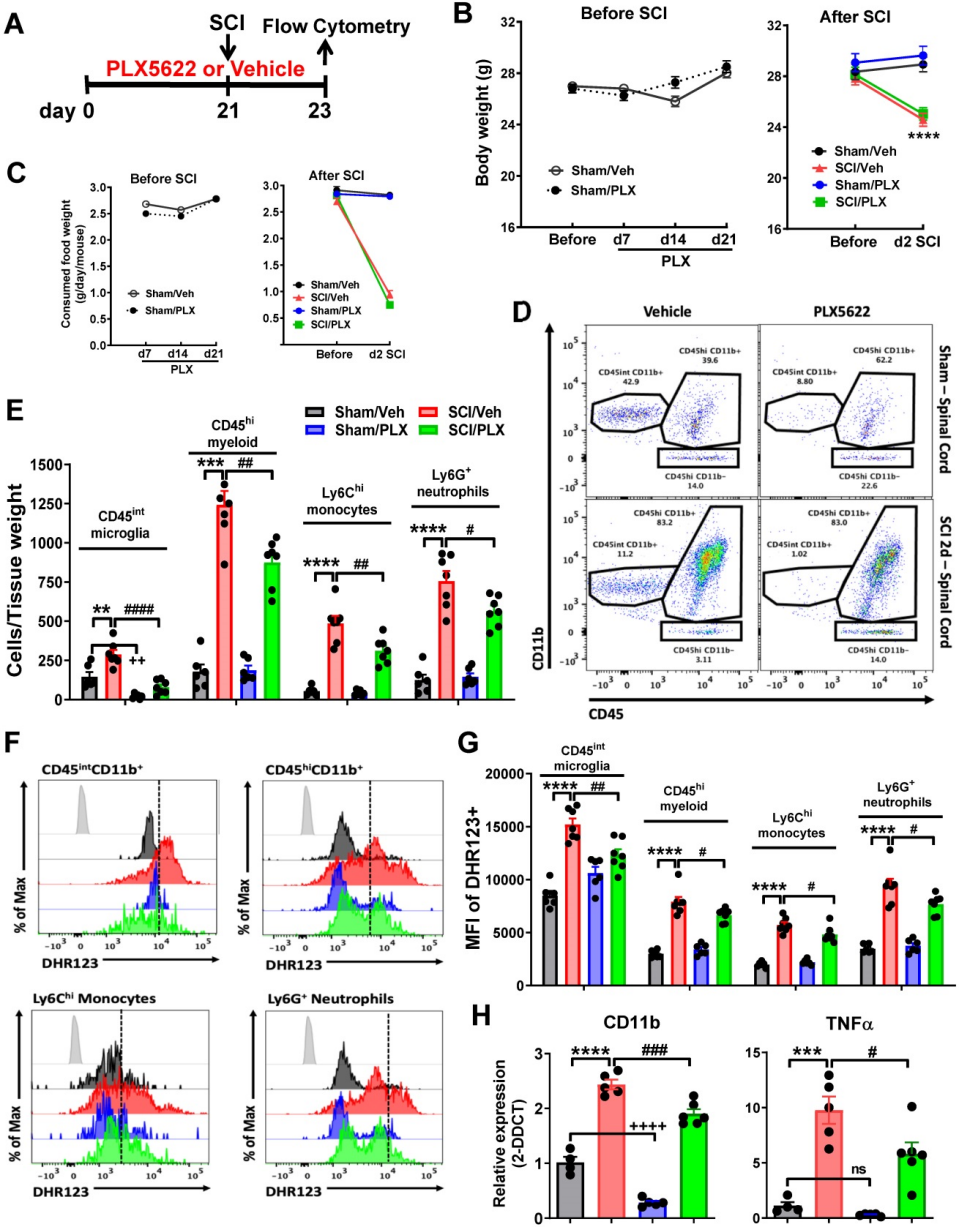
** Pre-depletion of microglia with PLX5622 reduces infiltrating cells and reactive oxygen species (ROS) production after SCI.** (**A**) PLX5622 diet or vehicle chow were fed to animals three weeks before injury and remained for 2 days after injury. (**B**) Body weight was recorded both prior and after injury. All injured mice appeared significant loss of body weight. PLX had no effects on animal weight both prior and after injury. n = 30 mice/group before SCI and 12 (Sham/Veh), 12 (Sham/PLX), 18 (SCI/Veh), and 18 (SCI/PLX) mice after SCI. (**C**) Food intake was recorded both prior and after injury. In all injured mice, food consumption decreased significantly. PLX had no effects on food consumption both prior and after injury. n = 15 mice/group (Before injury) and 10 (Sham/Veh), 10 (Sham/PLX), 15 (SCI/Veh), and 15 (SCI/PLX) mice (After SCI). (**D**) Representative dot plots show the relative immune cell composition in the impact site of the spinal cord at two days post-injury. (**E**) The number of living CD45^int^CD11b^+^Ly6C^-^ microglia, CD45^hi^CD11b^+^ infiltrating myeloid cells, CD45^hi^CD11b^+^Ly6C^+^Ly6G^-^ monocytes, and CD45^hi^CD11b^+^Ly6C^+^Ly6G^+^ neutrophils are quantified. n = 6 (Sham/Veh), 6 (Sham/PLX), 7 (SCI/Veh), and 7 (SCI/PLX). (**F**) Representative histograms show the relative production of ROS in microglia, bulk infiltrating myeloid cells, monocytes, and neutrophils as measured by DHR123. (**G**) The mean fluorescence intensity (MFI) of dihydrorhodamine 123 (DHR123) was quantified for all cells. For all flow cytometry experiments. n = 6 (Sham/Veh), 6 (Sham/PLX), 7 (SCI/Veh), and 7 (SCI/PLX). (**H**) Quantification of the myeloid marker CD11b and the pro-inflammatory cytokine TNFα by qPCR analysis at 2 days post-injury. Total RNA was extracted from sham or injured spinal cord. n = 4 (Sham/Veh), 5 (Sham/PLX), 5 (SCI/Veh), and 6 (SCI/PLX). **p < 0.01, ***p < 0.001, ****p < 0.0001, ++++*p <* 0.0001, vs. Sham/Veh group. #*p <* 0.05, ##*p <* 0.01, ##*p <* 0.001, ####*p <* 0.0001, vs. SCI/Veh group. 2-way ANOVA following Tukey's multiple comparisons test.

**Figure 2:**
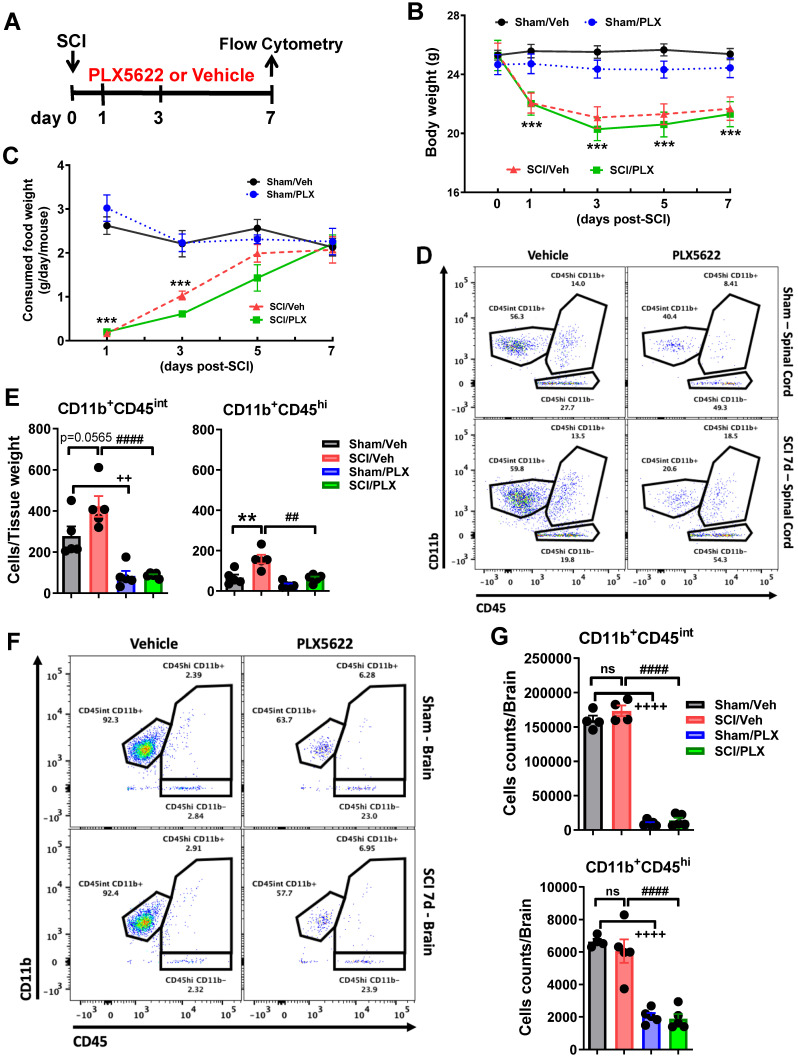
** PLX5622 treatment, when post-injury, reduces microglia and infiltrating cells in both intraspinal cord and brain at 7 days after SCI.** (**A**) PLX5622 diet or vehicle chow were fed to animals on day 1 post-injury and maintained up to 7 days. (**B-C**) Body weight and food intake were recorded before and 1, 3, 5, 7 days after injury. All injured mice displayed a significant decrease in both body weight and food consumption. PLX had no effects on animal weight and food intake after injury. n = 5 mice/group, ***p < 0.001, vs. Sham/Veh group. 2-way ANOVA with repeated measurements following Sidak's multiple comparisons test. (**D**) Representative dots plots show the relative immune cell composition in the impact site of the spinal cord at 7 days post-injury. (**E**) The number of living CD45^int^CD11b^+^Ly6C^-^ microglia (left) and CD45^hi^CD11b^+^ infiltrating cells (right) in each group is shown, n = 5 mice/group. (**F**) Representative dot plots depict the composition of immune cells in the brain after SCI. (**G**) The number of brain-resident microglia (top) and infiltrating myeloid cells (bottom) is quantified, n = 4-5 mice/group. *p < 0.01, ***p < 0.001, ++++*p <* 0.0001, vs. Sham/Veh group. ####*p <* 0.0001, vs. SCI/Veh group. 2-way ANOVA following Tukey's multiple comparisons test.

**Figure 3:**
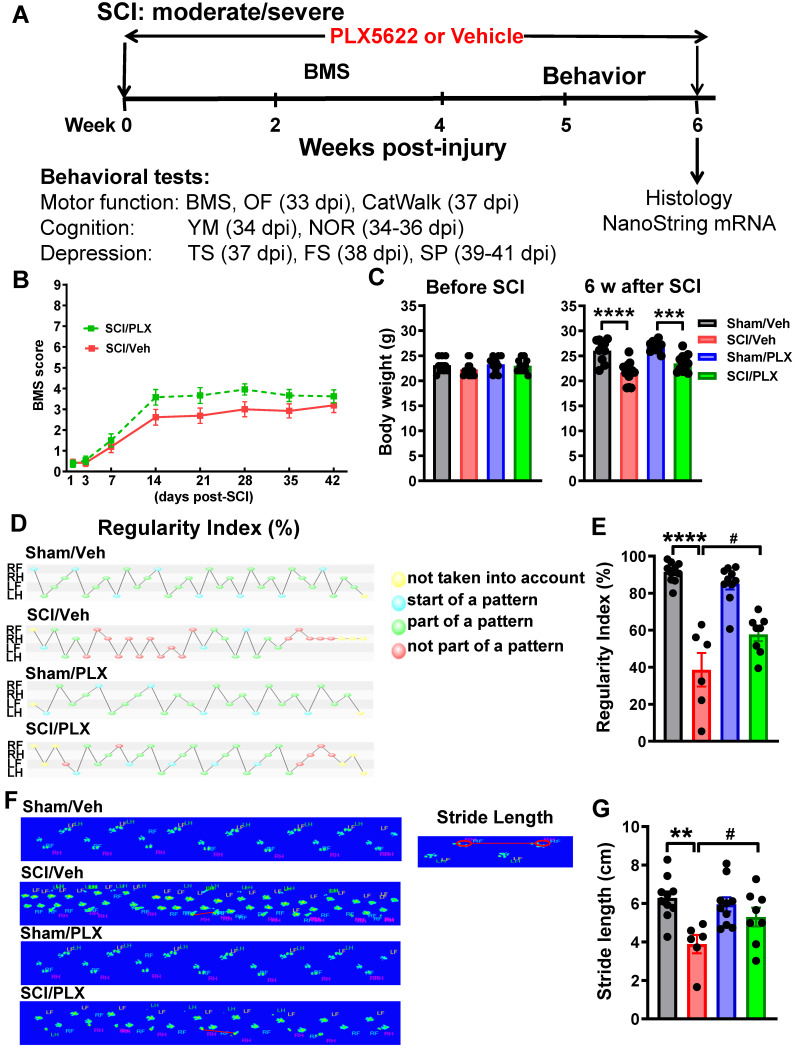
** Elimination of microglia by PLX5622 after SCI causes improvement in gait dynamics.** (**A**) Experimental timeline: adult male C57Bl/6 mice underwent moderate/severe contusion injury at T10. Right after the surgery, PLX5622 (1200 ppm) or Vehicle chow was fed to the mice for the remainder of the study. BMS was recorded on days 1, 3, and weekly up to 6 weeks post-injury. Beginning 5 weeks post-injury, all mice underwent a battery of neurobehavioral tasks [Motor function: open field (OF, 33 days post-injury, dpi), CatWalk (37 dpi); Cognitive function: Y maze (YM, 34 dpi), novel object recognition (NOR, 34-36 dpi); Depressive-like behavior: tail suspension (TS, 37 dpi), forced swim (FS, 38 dpi), sucrose preference (SP, 39-41 dpi)]. By the 6 weeks post-injury, injured spinal cord and cerebral cortex tissue were collected for nanostring analysis. (**B**) BMS. No significant differences were found in general locomotor recovery on the BMS. n = 13 (SCI/Veh) and 12 (SCI/PLX) mice (2-way ANOVA with repeated measurements following Bonferroni's multiple comparisons test). (**C**) Mouse body weight prior and after injury [n = 10 (Sham/Veh), 13 (SCI/Veh), 10 (Sham/PLX), and 12 (SCI/PLX)]. ***p < 0.001, ****p < 0.001 vs. Sham group. 2-way ANOVA following Tukey's multiple comparisons test. (**D-G**) Gait analysis was performed using the CatWalk apparatus. SCI mice capable of placing the plantar surface of the hindpaws on a flat surface were selected at 6 weeks after SCI. These parameters include step sequence regularity index (D,E) and stride length of the hind limbs (F,G). Representative images of regularity index (D) illustrate that the beginning of a step cycle is shown here in blue and subsequent steps within the cycle that fit a standard pattern shown in green, while irregular steps appear in red. RF, RH, LF, and LH represent right forepaw, right hindpaw, left forepaw, and left hindpaw, respectively. F illustrates representative images of stride length. [n = 10 (Sham/Veh), 6 (SCI/Veh), 10 (Sham/PLX), and 8 (SCI/PLX)]. **p < 0.01, ****p < 0.0001, vs. Sham/Veh; #*p <* 0.05, vs SCI/Veh. 2-way ANOVA following Tukey's multiple comparisons test.

CSF1R inhibitor Plexxikon (PLX) 5622 was provided by Plexxikon Inc. (Berkley, CA) and formulated in AIN-76A rodent chow by Research Diets Inc. (New Brunswick, NJ) at a concentration of 1200 ppm 42. According to the provider, the specialized diet was stored in a 4 °C refrigerator. Mice were provided ad libitum access to PLX5622 diet or standard AIN-76A chow as vehicle control. Detailed PLX treatment protocols were indicated in Figures 1A, 2A, 3A, and 5A.

### Experimental design

#### Study 1

To determine the effects of microglia depletion on CNS-infiltrating cells after SCI, C57BL/6 mice were fed PLX5622 diet (n = 30 mice) or vehicle chow (n = 30 mice) starting 3 weeks before SCI until time of sacrifice. Body weights and food intake were recorded before and after chow feeding or SCI. At 2 days post-injury, mice were anesthetized (100 mg/kg sodium pentobarbital, I.P.) and blood (200 μL) was drawn by cardiac puncture with heparinized needles and leukocyte composition was determined. After transcardially perfusion with ice-cold 0.9% saline (40 mL), the fresh ~1 cm of spinal cord tissue surrounding the epicenter of the lesion site was dissected and the cell suspension was prepared for flow cytometry assays [n = 6 (Sham/Veh), 7 (SCI/Veh), 6 (Sham/PLX), and 7 (SCI/PLX)]. A subset of the cell suspension was frozen on liquid nitrogen for RNA extraction [n = 4 (Sham/Veh, derived from 6 mice), 5 (SCI/Veh, derived from 10 mice), 5 (Sham/PLX, derived from 6 mice), and 6 (SCI/PLX, derived from 10 mice)]. A total of 2 mice were excluded because of poor tissue dissection.

#### Study 2

To test whether microglial depletion after injury affects the acute inflammatory response, C57BL/6 mice (n = 5 mice/group) were fed PLX5622 diet or vehicle chow starting on the date of the injury until the time of sacrifice. Body weights and food intake were recorded before and after SCI. At 7 days post-injury, mice were anesthetized (100 mg/kg sodium pentobarbital, I.P.) and transcardially perfused with ice-cold 0.9% saline (40 mL). The fresh ~1 cm of spinal cord tissue surrounding the epicenter of the lesion site or brain tissue was dissected for flow cytometry assays.

#### Study 3

To investigate whether microglial depletion after injury affects neurological function after SCI, C57BL/6 male mice were subjected to either moderate/severe (60 kdyn) contusion injury or sham surgery. At the date of injury, animals were placed on PLX5622 or Vehicle chow for up to 6 weeks [n = 10 (Sham/Veh), 15 (SCI/Veh), 10 (Sham/PLX), and 15 (SCI/PLX)]. All animals underwent locomotor function testing (BMS) on 1, 3, 7, 14, 21, 28, 35, 42 days post-injury (dpi). Beginning 5 weeks post-injury, all mice underwent a battery of neurobehavioral tasks [Motor function: open field (OF, 33 days post-injury, dpi), CatWalk (37 dpi); Cognitive function: Y maze (YM, 34 dpi), novel object recognition (NOR, 34-36 dpi); Depressive-like behavior: tail suspension (TS, 37 dpi), forced swim (FS, 38 dpi), sucrose preference (SP, 39-41 dpi)]. At 6 weeks post-injury, mice were anesthetized with isoflurane and transcardially perfused with ice-cold 0.9% saline (100 mL). Injured spinal cord and dissected cerebral cortex tissue were collected for NanoString analysis. A total of 5 mice were excluded because of autophagia (4 mice) or bladder infection (1 mouse).

#### Study 4

To determine whether the effect of PLX5622 on locomotor function was injury severity-dependent, Cx3xr1-GFP male mice were subjected to either severe (70 kdyn) contusion injury or sham surgery. At the date of injury, animals were placed on PLX5622 or Vehicle chow for up to 6 weeks [n = 10 (Sham/Veh), 15 (SCI/Veh), 10 (Sham/PLX), and 15 (SCI/PLX)]. All animals underwent locomotor function testing (BMS) on 1, 3, 7, 14, 21, 28, 35, 42 days post-injury (dpi). By 6 weeks post-injury, mice were anesthetized with isoflurane and transcardially perfused with ice-cold 0.9% saline (100 mL), or followed by 300 mL of 4% paraformaldehyde (PFA). Injured spinal cord and brain tissue were collected and processed for NanoString analysis and histological outcome measures. A total of 8 mice were excluded because of mild injury (1 mouse), autophagia or autotomy (4 mice), and bladder or skin infection (3 mice).

### Flow cytometry assays

Mice were perfused with 40 mL of cold PBS and the fresh ~1 cm of spinal cord tissue surrounding the epicenter of the lesion site was weighed to control for any variation and normalize cell counts. The intact brain was isolated from the same mice. The olfactory bulb and cerebellum were removed, brains were halved along the interhemispheric fissure, and the left hemisphere was placed separately in complete Roswell Park Memorial Institute (RPMI) 1640 (Cat# 22400105, Invitrogen) medium and mechanically and enzymatically digested in collagenase/dispase (Cat# 10269638001, 1 mg/mL; Roche Diagnostics), papain (Cat# LS003119, 5 U/mL; Worthington Biochemical), 0.5 M EDTA (Cat# 15575020, 1:1000; Invitrogen), and DNAse I (Cat# 10104159001, 10 mg/mL; Roche Diagnostics) for 1 h at 37 °C on a shaking incubator (200 rpm). The cell suspension was washed twice with RPMI, filtered through a 70 μm filter, and RPMI was added to a final volume of 5 mL/spinal cord segment and 5 ml/brain hemisphere and kept on ice. Cells were then transferred into FACS tubes and washed with FACS buffer. Cells were then incubated with Fc Block (Cat# 101320, Clone: 93; Biolegend) for 10 min on ice, and stained for the following surface antigens: CD45-eF450 (Cat# 48-0451-82, Clone: 30-F11; eBioscience), CD11b-APC/Fire™750 (Cat# 101262, Clone: M1/70; Biolegend), Ly6C-APC (Cat# 128016, Clone: HK1.4; Biolegend), Ly6G-PE (Cat# 127607, Clone: 1A8; Biolegend), and Zombie Aqua fixable viability dye (Cat# 423102, Biolegend). Cells were then washed in FACS buffer, fixed in 2% paraformaldehyde for 10 min, and washed once more prior to adding 500 µL FACS buffer. Relative changes in ROS production were measured using the cell-permeant fluorescent dye probe dihydrorhodamine 123 (DHR123, 1:500; Invitrogen) as described previously 54.

Data were acquired on a BD LSRFortessa cytometer using FACSDiva 6.0 (BD Biosciences) and analyzed using FlowJo (Treestar Inc.). At least 5-10 million events were collected for each sample. Countbright™ Absolute Counting Beads (Invitrogen) were used to estimate cell counts per the manufacturer's instructions. Data were expressed as either cells/mg tissue weight or back-calculated to estimate total counts/hemisphere. Leukocytes were first gated using a splenocyte reference (SSC-A vs FSC-A). Singlets were gated (FSC-H vs FSC-W), and live cells were gated based on Zombie Aqua exclusion (SSC-A vs Zombie Aqua-Bv510). The cell permeant nuclear stain Draq5 (Cat# 424101, 1:500; Biolegend) was used in reference CNS samples to help delineate the leukocyte and singlet gates. Resident microglia were identified as the CD45^int^ CD11b^+^Ly6C^-^ population, and CNS-infiltrating leukocytes were identified as CD45^hi^CD11b^+^ myeloid cells or CD45^hi^CD11b^-^ lymphocytes. Within the CD45^hi^ myeloid subset, monocytes were identified as Ly6C^hi^Ly6G^-^ and neutrophils, Ly6C^+^Ly6G^+^ 55 (see **Figure S1**).

### Quantitative PCR

Total RNA was extracted from the spinal cord (~5 mm surrounding the epicenter of the lesion site) of sham and SCI mice with a miRNeasy isolation kit (Cat# 74104, Qiagen). Complementary DNA (cDNA) was synthesized by a Verso cDNA RT kit (Cat# AB1453B, Thermo Scientific) per the manufacturer's protocol. Real-time PCR for target mRNAs was performed using TaqMan gene expression assays for Itgam (CD11b), Mm00434455_m1, TNFα, Mm00443258_m1; Csfr1, Mm01266652_m1; P2ry12, Mm00446026_m1; Trem2, Mm04209424_g1; Mfge8, Mm00500549_m1; Casp1, Mm00438023_m1; Casp6, Mm01321726_g1; Casp8, Mm01255716_m1; Bax, Mm00432051_m1; Bcl2, Mm00477631_m1; Gfap, Mm01253033_m1; Il-1a, Mm00439620_m1; Lrrc25, Mm00462019_m1; Gbp2, Mm00494576_g1; Osmr, Mm01307326_m1; Entpd2, Mm00515450_m1; Vim, Mm01333430_m1; C4a, Mm01132415_g1; GAPDH, Mm99999915_g1 (Applied Biosystems, Carlsbad, CA] on an QuantStudio™ 5 Real-Time PCR System (Applied Biosystems). qPCR reactions were run in duplicates to eliminate mistakes of pipetting, which was composed of 3 stages, 50 °C for 2 min, 95 °C for 10 s for each cycle (denaturation), and finally, the transcription step at 60 °C for1 min. Gene expression was normalized by GAPDH and compared to the control sample to determine relative expression values by the 2-ΔΔCt method.

### Basso mouse scale (BMS) for locomotion

Mice were placed in a flat, enclosed surface (diameter = 100 cm) and observed for 4 min by two trained observers using the Basso mouse scale (BMS) for locomotion 56. Animals were rated on a scale of 0-9: 0 being complete paralysis, and 9 being normal locomotion based on hind limb joint movement, weight support, plantar stepping, and coordination. Mice were tested for BMS scores on day 1 after injury and weekly thereafter for up to 6 weeks.

### Spontaneous motor activity

The open field (OF) test was used to measure locomotor activity on 33 days post-injury (dpi). Mouse was individually placed in a corner facing the wall of the open-field chamber (22.5 cm × 22.5 cm) and allowed to freely explore the chamber for 5 minutes. The distance travelled, average speed, time immobile vs mobile and rearing/elongation behavior were recorded by Any-Maze software (Stoelting Co.).

### CatWalk XT automated gait analysis

Motor coordination was performed and analyzed using the CatwalkXT automated system (Noldus; RRID:SCR_004074) 54. In a dark (unlit) room, animals were placed on the walkway and allowed to traverse from one end to the other. Direct contact between the paw and glass surface results in light reflection in the form of illuminated footprints. Footprint images were video-recorded by a camera positioned under the walkway. The images from each trial were converted into digital signature and processed using CatWalk XT 9.1 software with a minimum threshold set at 80 (a.u. ranging from 0 to 225). Following footprint identification and labeling, data pertaining to static and dynamic gait parameters are generated for each trial. The mean scores from 3 consecutive trials (per animal/time points) are analyzed for statistical significance. Trials in which the animal stopped partway across or turned around during a run were excluded from analysis.

### Y-maze test

The Y-maze was performed at 34 dpi to test spatial working memory in mice, as previously described 54. The Y-maze (Stoelting Co.) consisted of three identical arms (A, B, C). During testing, one arm was randomly selected as the “start” arm, and the mouse was placed in the maze freely for 5 min. Arm entries were recorded and an alternation was designated when the mouse entered three different arms consecutively. The percentage of alternation was calculated as follows: total alternations x 100/(total arm entries - 2). If a mouse scored significantly >50% alternations (the chance level for choosing the unfamiliar arm), this was indicative of spatial working memory.

### Novel object recognition (NOR)

NOR was performed on 34-36 dpi to assess non-spatial hippocampal-mediated memory, as previously described 54. On day 1, mice were individually placed in an open field (22.5 cm X 22.5 cm) for 5 min free moving for habituation. Next day, mice were placed in an open field where two identical objects were placed near the left and right corners of the chamber for training (sample phase). After 24 h, object recognition was tested by substituting a novel object for a familiar training object (the novel object location was counterbalanced across mice). Time spent with two identical objects was recorded; because mice inherently prefer to explore novel objects, a preference for the novel object (more time than chance (15 s) spent with the novel object) indicates intact memory for the familiar object.

### Tail-suspension test

The tail-suspension (TS) test assesses depression-like behavior in mice and is based on the observation that mice develop an immobile posture when placed in an inescapable hemodynamic stress of being hung by their tail. The TS was performed on 37 dpi as described previously 15. Each mouse was suspended at a height of 28 cm using 3 M adhesive tape. The tip of the mouse tail was not wrapped around the rod while being suspended. The duration of immobility was recorded throughout the 5 min test period. The definition of immobility is passive hanging and complete motionlessness. Foam padding (3-inch deep) was placed under the beam in case animals fall from the beam during the experiment.

### Forced swim test

Forced swim test (FS) is one of the most commonly used assays for the study of depressive-like behavior in rodents. On 38 dpi, mice were placed in transparent plastic cylinder (45 cm high × 20 cm diameter) filled with water (23 ± 2 °C; 28 cm in depth) for 6 min 15. The duration of immobility was recorded.

### Sucrose preference test

Sucrose preference (SP) test is used as an indicator of anhedonia, which is present in some affective diseases, such as depression. On 39-41 dpi, we evaluated the mouse's interest in seeking a sweet rewarding drink relative to plain water 15. Failure to demonstrate a bias toward the sweetened drink indicates depression-like behavior. The saccharine was weighted, dissolved in 5 mL plain water and injected into hydrogel pack with syringe to get final 0.3% saccharine concentration. Initial weights of hydrogel pack containing plain water and saccharine solution were recorded. Two inserts were placed in each cage with plain water insert at the normal back position and saccharine water insert at the front position. 100 g food was added to each insert. The mouse was weighted and housed singly in each cage. After first 24 h, the water pouches, food, and mice were weighted and recorded. The insert with plain water and insert with saccharine water were rotated to avoid place preference. After second 24 h, the water pouches, food, and mice were weighted and recorded again. The sucrose preference was calculated by divided consumption of sweetened water by total consumption of water (sweetened water plus plain water).

### Tissue processing and histological analysis

Following animal perfusion with 4% paraformaldehyde, spinal cord segments containing the lesion area were dissected out, embedded, and cut into 20-μm-thick serial sections placed serially on set of 10 slides for 10 sets of slides. A representative slide from each set was then stained for myelin using Luxol fast blue (LFB) to determine the location of the lesion epicenter, defined as the section with the least amount of spared white matter (WM). Residual WM was also calculated for areas rostral and caudal to the lesion epicenter 57. Images were captured at ×2.5 magnification and analyzed using National Institutes of Health ImageJ software (RRID:SCR_003070). The threshold level of each 8-bit image was set to mark only LFB-positive tissue, and total LFB-positive area was calculated for each section. Sections spaced 1 mm apart from 5 mm caudal to 5 mm rostral the injury epicenter were stained with GFAP and DAB as the chromogen for lesion volume assessment. Quantification was based on the Cavalieri method using Stereoinvestigator Software (MBF Biosciences), as previously described 58. The lesion volume was quantified by outlining the missing tissue on the injured core using the Cavalieri estimator with a grid spacing of 0.1 mm.

### Immunofluorescence imaging and acquisition

Coronal spinal cord sections from CX3CR1-GFP mice were applied for immunohistochemistry (IHC) staining followed procedures described. Sections were blocked with 5% goat or donkey serum diluted in 0.3% Triton X-100 solution and cell nuclei were labeled with 4',6-diamidino-2-phenylinodole (DAPI, Sigma-Aldrich) and slides were cover-slipped with an anti-fade medium (Hydromount, National Diagonistics). All images were acquired using a fluorescent Nikon Ti-E inverted microscope, at 20X (CFI Plan APO VC 20X NA 0.75 WD 1 mm) magnification and the background of each image was subtracted using background ROI 15. All images were quantified using Elements: nuclei were identified using Spot Detection algorithm; cells positive for GFP were identified using Detect Regional Maxima algorithm, followed by global thresholding. The number of GFP^+^ cells was normalized to the total imaged area (mm^2^). Data from all images from one region in each mouse was summed up and used for final statistical analysis. At least 500-1000 cells were quantified per mouse per experiment. The images were acquired 0.4 mm rostral or caudal to the epicenter, with n = 2-4 images per location (whiter matter and grey matter) from four to five sections per mouse. Representative Cx3cr1-GFP+ images were obtained by tile scan using a Nikon A1 Laser Confocal microscope. In addition, the following primary antibodies were used: rabbit anti-GFAP (1:200, Cat# PA5-16291, Invitrogen); rat anti-F4/80 (1:600, Cat# ab6640, Abcam). Images were acquired by tile scan using a Nikon A1 Laser Confocal microscope. For GFAP+ area, images were acquired from 1.4 mm rostral or caudal to the epicenter by tile scan using a Nikon A1 Laser Confocal microscope.

### Neuronal density

Brain coronal sections at 60 µm thickness were stained with cresyl violet (FD NeuroTechnologies), and the optical fractionator method of stereology using Stereoinvestigator Software (MBF Biosciences) was employed 16. Neurons in motor cortex (M1, M2), thalamus [the ventral posterolateral nucleus (VPL), the ventral posteromedial nucleus (VPM), the posterior thalamic nucleus (PO), and anterior nucleus], hippocampal cornu Ammonis 1 (CA1), CA2/3, and the dentate gyrus (DG) regions were characterized according to previously described methods. Approximate positions of relevant anatomical structures were followed by mouse brain atlas overlay (http://www.mbl.org/atlas170/atlas170_frame.html). A total of 6 sections were analyzed for each animal and the total number of surviving neurons in each field was divided by volume of that region of interest to obtain an end result of counts/mm^3^, which reflects cellular density of neurons in the region.

### Samples preparation and NanoString analysis

Total RNA was extracted from previously flash-frozen spinal cord tissue (~5 mm) surrounding the epicenter of the lesion site as well as dissected cerebral cortex (including motor cortex and somatosensory cortex) using Qiagen RNA extraction kit. Total RNA (20 ng/µL) was run on an nCounter® Mouse Neuroinflammation v1.0 panel (NanoString Technologies, Seattle, WA) to simultaneously measure RNA transcript counts for 757 genes and 13 housekeeping genes. Genes were categorized by NanoString into 23 pathway annotations across three themes (number of pathways in parentheses): Immunity and Inflammation (6); Neurobiology and Neuropathology (13); and Metabolism and Stress (4).

Sample gene transcript counts were normalized prior to downstream analysis - unless specifically noted elsewhere - with NanoString's nSolver software Version 4.0 that uses a geoNorm algorithm to identify stable housekeeping genes for normalization 59. All statistical analysis of NanoString data was performed in the R language using RStudio Version 1.2.5033. Principal component analysis (PCA) was performed with the “prcomp()” function in R. Differential expression analysis between paired groups was performed in R with the “NanoStringDiff” package Version 1.18.0, which performs normalization using the raw gene transcript counts, positive and negative controls, and housekeeping gene transcript data provided by NanoString as described 60. The four pairwise comparisons were as follows and described in current study as: (1) Sham/PLX *vs.* Sham/Veh - PLX Comparison 1; (2) SCI/Veh *vs.* Sham/Veh - Injury Comparison 2; (3) SCI/PLX *vs.* SCI/Veh - PLX Comparison 3; and (4) SCI/PLX *vs.* Sham/PLX - Injury Comparison 4. All comparisons “Group 1 *vs.* Group 2” were interpreted as “Group 1 relative to Group 2” in the text and figures. NanoStringDiff uses the Benjamini-Hochberg method for false discovery rate (FDR) correction, and an adjusted p-value less than 0.05 was used to identify differentially expressed (DE) genes in each comparison 60 except for the SCI/Veh *vs.* Sham/Veh comparison in the cortex (non-adjusted *p*-value < 0.05). Subsets of DE genes displayed as heatmaps were normalized across samples as *z*-scores and then averaged to a single value per group before plotting using GraphPad Prism Version 8.4.2.

Protein-protein interaction (PPI) network maps for gene sets were generated through the STRING database Version 11.0 61. Network edges depict the level of confidence (thicker lines: higher confidence) in the interaction based on literature evidence curated by STRING. In some cases, PPI maps were imported into Cytoscape Version 3.8.0 for further visual enhancements of the network 62. Pathway diagrams were constructed using Cytoscape and annotated by direct binding or regulatory actions experimentally determined through literature curated by Elsevier's Pathway Studio Version 12.3.0.16 63.

### Statistical analysis

Quantitative data were expressed as mean ± standard errors of the mean (SEM). All statistical analyses were conducted by using the GraphPad Prism Program, Version 3.02 for Windows (GraphPad Software; RRID:SCR_002798). BMS scores were analyzed using two-way ANOVA with repeated measures followed by Sidak's multiple comparisons post hoc test. For multiple comparisons, one-way or two-way ANOVA were performed followed by Tukey's multiple comparisons post hoc test for parametric (normality and equal variance passed) data. Stereological data for lesion volume was analyzed using a Student *t* test. Statistical analysis in each assay was detailed in figure legends. A *p* value of < 0.05 was considered statistically significant.

## Results

### Depletion of microglia with CSF1R inhibitor reduces infiltrating cells and ROS production after SCI

Traumatic injury to spinal cord elicits an acute intraspinal inflammatory responses including resident microglial reactivation and infiltrating blood leukocytes. The latter is neurotoxic to the CNS tissues. To determine the effects of microglia depletion on infiltrating cells after SCI, CSF1R inhibitor PLX5622 diet or vehicle chow was fed to the animals three weeks before injury and remained for the during of the study (**Figure 1A**). Injury biomechanics indicated that there was no difference in injury forces, displacement, and BMS score (at 24 h post-injury) between SCI/Veh and SCI/PLX groups (**Figure S1A**). Although all injured mice appeared significant loss of body weight and food consumption, PLX had no effects on animal weight (**Figure 1B**) and food intake (**Figure 1C**) both prior and after injury. At 2 days post-injury, flow cytometry was used to examine neuroinflammation in the impact site. Representative dot plots illustrating our gating strategy for identifying CD45^int^CD11b^+^Ly6C^-^ CNS-resident microglia and CD45^hi^CD11b^+^ infiltrating myeloid cells are shown (**Figure S1B**). A splenocyte reference and Draq5 staining were used to identify single nucleated living leukocyte populations in CNS tissue. Flow cytometry analysis revealed that PLX drug significantly reduced the number of CD45^int^CD11b^+^ microglia in the spinal cord under sham conditions (**Figure 1D-E**). After injury, microglia counts were increased in both groups, albeit significantly more so in the vehicle treated group. Continuous PLX treatment significantly attenuated the number of CD45^hi^CD11b^+^ infiltrating myeloid cells compared to injured control (**Figure 1D-E**). Reductions in both monocyte and neutrophil invasion were seen. Given the detrimental role of oxidative stress in sterile CNS injury and inflammation we next assessed the level of ROS production in immune cells using DHR123. Although there were significantly fewer microglia during PLX treatment, microglial ROS levels were significantly higher after SCI in vehicle treated groups (**Figure 1F-G**). ROS production by infiltrating monocytes and neutrophils was relatively decreased during PLX5622 treatment compared to SCI/Veh control (**Figure 1F-G**). In addition, we examined whether systemic administration of PLX5622 affected circulating leukocyte populations after SCI. At 2 days post-injury, SCI significantly increased circulating CD45^hi^ monocytes and Ly6G^+^ neutrophils, but reduced CD11b^-^CD45^hi^ lymphocytes. However, SCI mice that had been on PLX5622 diet (for a total of 3 weeks) did not show dysregulation of blood cell numbers (**Figure S2**). Tissue-level gene expression of the myeloid marker CD11b confirmed PLX-mediated depletion of spinal cord microglia (**Figure 1H**). This marker and the pro-inflammatory cytokine TNFα were up regulated after injury, albeit significantly less so after PLX treatment compared to injured control (**Figure 1H**). Together these findings show that elimination of microglia by pre-treatment with PLX reduces the severity of neuroinflammation following acute SCI.

To test whether microglial depletion after injury affected the resolution of the acute inflammatory response, PLX5622 diet or vehicle chow was fed to the animals on day 1 post-injury and maintained up to 7 days (**Figure 2A**). Injury biomechanics indicated that there was no difference in injury forces and displacement between SCI/Veh and SCI/PLX groups (**Figure S3A**). No significant differences were observed between two injured groups in the BMS during the first week post-injury (**Figure S3B**). Although all injured mice appeared significant loss of body weight and food consumption, PLX had no effects on animal weight (**Figure 2B**) and food intake (**Figure 2C**) after injury. At 7 days post-injury, flow cytometry was used to examine the cellular correlates of neuroinflammation. Consistent with our previous results 58, we found that continuous PLX treatment significantly reduced both microglia and infiltrating myeloid counts in the impact site compared to injured control (**Figure 2D-E**). Because our group has previously reported 14-16 brain-related changes following SCI we also evaluated the effect of PLX in the brain. Interestingly, while microglia counts were markedly decreased in the brain during drug treatment as predicted, we also observed significant reductions in other myeloid cell populations that reside in the brain under normal conditions (**Figure 2F-G**). These results demonstrate that microglia promote leukocyte infiltration in the spinal cord following traumatic injury.

### Long-term elimination of microglia with PLX5622 improves neurological functional recovery after SCI

We next investigated whether reduced microglia and infiltrating monocytes affect neurological function after SCI. Following clinical relevant treatment paradigm, when after injury, PLX5622 diet or vehicle chow was fed to the animals on day 1 post-injury up to 6 weeks. Comprehensive motor, cognitive, and depressive-like functional testing was performed from 5 to 6 weeks post-injury (**Figure 3A**). Hindlimb locomotor function was tested in the open field on days 1, 3, and weekly up to 6 weeks post-injury. At 24 h after SCI, all injury mice had a BMS score of 0 or 1, indicating complete loss of motor function (**Figure 3B**). No significant differences were observed between drug- or vehicle-treated groups in gross motor function in the BMS. Although there were no differences of mice body weight prior injury among four groups, moderate/severe injury in male mice caused significant body weight loss (**Figure 3C**). However, six weeks of PLX treatment had no effect on animal weight between both sham or both injury groups.

To detect more refined kinematic properties of locomotion beyond that recognized by BMS scores, we quantified gait dynamics using the CatWalk apparatus. SCI mice capable of placing the plantar surface of the hindpaws on a flat surface were selected at 6 weeks after SCI. Stepping pattern was a parameter for evaluation of overall motor coordination, which tracks step sequencing in a step cycle. A percentage of normal stepping (regularity index) ideally reaches close to 100% in healthy animals 64. In response to SCI, the regularity index in vehicle-treated mice was reduced by nearly half compared with Sham/Veh animals. Remarkably, SCI/PLX mice appeared significantly restored step sequencing, approaching 60% normal stepping (**Figure 3D-E**, F(1,30)=8.903, *p=*0.0056). Stride length, categorized as the total distance between the successive placement of the same paw, was significantly reduced in all injured mice compared with sham animals. PLX treatment in SCI mice significantly improved the placement of the hindpaws compared with SCI/Veh animals (**Figure 3F-G,** F(1,29)=6.523, *p=*0.0162). Print position, defined as the distance between the hindpaw and forepaw of the same side, scores typically approaching 0-1 cm in healthy mice. All injured mice showed dramatically increased print position (**Figure S4A**). Print area, defined as the surface area of the complete print, is by definition at least as large as max contact area. SCI significantly reduced print area as well as max contact area of the hindpaws compared with sham controls, suggesting an increase in spontaneous pain-type behaviors in the automated Cat-Walk. There were no apparent differences in the SCI mice between PLX and Vehicle groups with regard to print position, print area, and max contact area of the hindpaws. PLX treatment also had no effect on motor coordination in sham mice.

In addition, spontaneous motor activity was recorded in the open-field chamber at 5 weeks post-injury by computer-based Any-Maze automated video tracking system 15. SCI resulted in a significantly reduced distance traveled and walking speed, compared with sham mice (**Figure S4B**). However, elimination of microglia by PLX treatment did not improve overall animal motor activity. We also did not observe the differences of percentage of distance inside zone among four groups.

**Figure 4:**
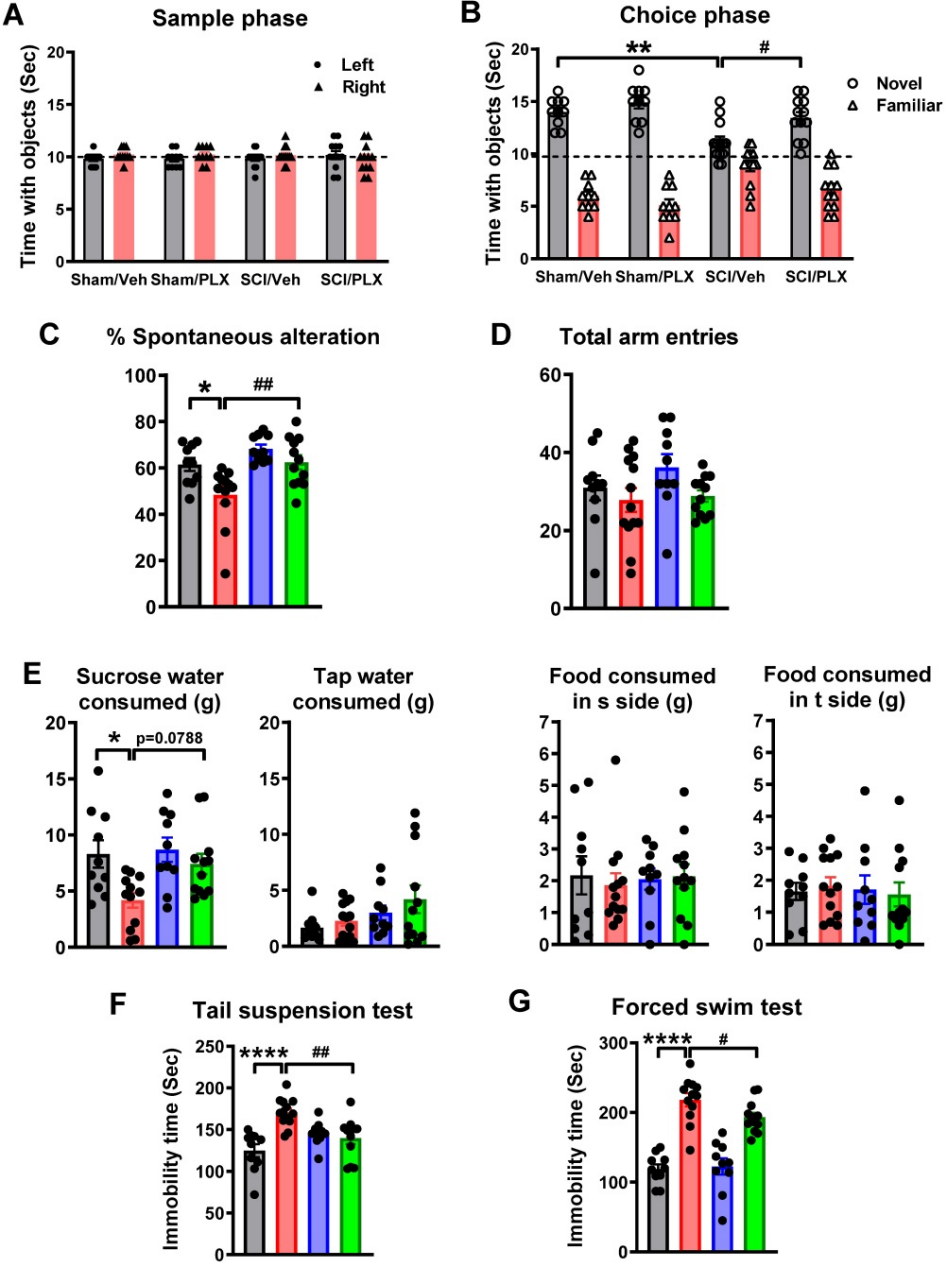
** Systemic depletion of microglia by PLX5622 improves cognitive and depressive-like behavior after SCI.** (**A,B**) Novel object recognition (NOR) test. All sham and SCI mice spent equal time with the two identical objects during the training (sample) phase. SCI mice spent significantly less time with the novel object. SCI/PLX mice showed increased time interacting with the novel object. (**C,D**) Y maze spontaneous alteration test. SCI-mediated changes in the percentage of spontaneous alterations and total number of arm entries are shown. (**E**) Sucrose preference test (SP). The SP is calculated by divided consumption of sweetened water (0.5% saccharine) by total consumption of water (sweetened water plus plain water). The food reference also is calculated as a control to demonstrate that mice do not show a place preference. SCI mice showed significantly reduced sweet water consumption without a change in food consumption. PLX treatment increased sucrose intake in SCI mice (*p=*0.0788) compared with SCI/Veh animals. (**F**) Tail suspension test. SCI-mediated increased immobility times was significantly reduced in SCI/PLX group. (**G**) Forced swim test. SCI results in increases in immobility in unescapable water cylinder. PLX treatment significantly reduces immobility times, compared with the SCI/Veh group. [n = 10 (Sham/Veh), 13 (SCI/Veh), 10 (Sham/PLX), and 12 (SCI/PLX)]. *p < 0.05, **p < 0.01, ****p < 0.0001, vs. Sham/Veh; #*p <* 0.05, ##*p <* 0.01, vs SCI/Veh. 2-way ANOVA following Tukey's multiple comparisons test.

To explore the long-term effects of microglial depletion on cognitive function and depressive-like behavior after SCI, we performed a variety of neurobehavioral tests that are less dependent on locomotion 15. Non-spatial recognition memory was evaluated by a NOR test at 6 weeks post-injury. During the training (sample) phase, sham and SCI mice spent equal time with the two identical objects (**Figure 4A**), indicating intact memory in the presence and absence of PLX. At 24 h after the training, SCI/Veh mice spent significantly less time with the novel object (**Figure 4B,** F(1,41)=8.929, *p=*0.0047). SCI/PLX mice showed increased time interacting with the novel object, suggesting improved short-term recognition memory and preference for novelty. No significant differences were observed between the Sham/Veh and Sham/PLX groups. Hippocampus-dependent spatial working memory was examined by the Y maze testing at 5 weeks post-injury. Both groups of sham mice showed approximately 70% spontaneous alteration, indicative of functional working memory (**Figure 4C,** F(1,40)=11.63, *p=*0.0015). SCI/Veh mice revealed a significant reduction in spontaneous alterations and no change in total entries (**Figure 4D**), indicative of impairment of spatial working memory. Elimination of microglia by PLX caused a significant improvement of spontaneous alteration compared with SCI/Veh animals.

To investigate whether microglial depletion after SCI causes changes in depressive-like behavior, we used an array of established tests 54. To reduce the potential confounding effects of motor function deficits in SCI mice, the sucrose preference test was evaluated at 6 weeks post-injury. Compared to sham animals, SCI mice showed significantly reduced sweet water consumption independent of tap water and food consumption, indicating increased anhedonia (**Figure 4E,** F(1,40)=7.795, *p=*0.0080). PLX treatment increased sucrose intake in SCI mice (F(1,40)=3.489, Tukey's multiple comparisons test, *p=*0.0788) compared with SCI/Veh animals. Immobility in tail-suspension and forced swim tests is interpreted as a symptom of learned helplessness and characteristic of depression in rodent subjects. As shown in **Figure 4F-G**, SCI mice displayed greater immobility at 6 weeks post-injury (F(1,41)=17.27, *p=*0.0002 for TS; F(1,39) = 115.6, *p <* 0.0001 for FS). SCI/PLX mice showed significant reduction in immobility times, compared with the SCI/Veh group (F(1,41)=10.77, *p=*0.0021 for TS; F(1,39)=4.606, *p=*0.0381 for FS).

Collectively, these data show that post-injury microglial depletion by PLX in male moderate/severe SCI mice displayed improvement in motor coordination, as well as in cognitive and depressive-like behavioral tasks. PLX treatment had no effect on neurological function tested in sham mice.

### Depletion of microglia after SCI reduces neurodegeneration in the brain sub-regions

**Figure 5:**
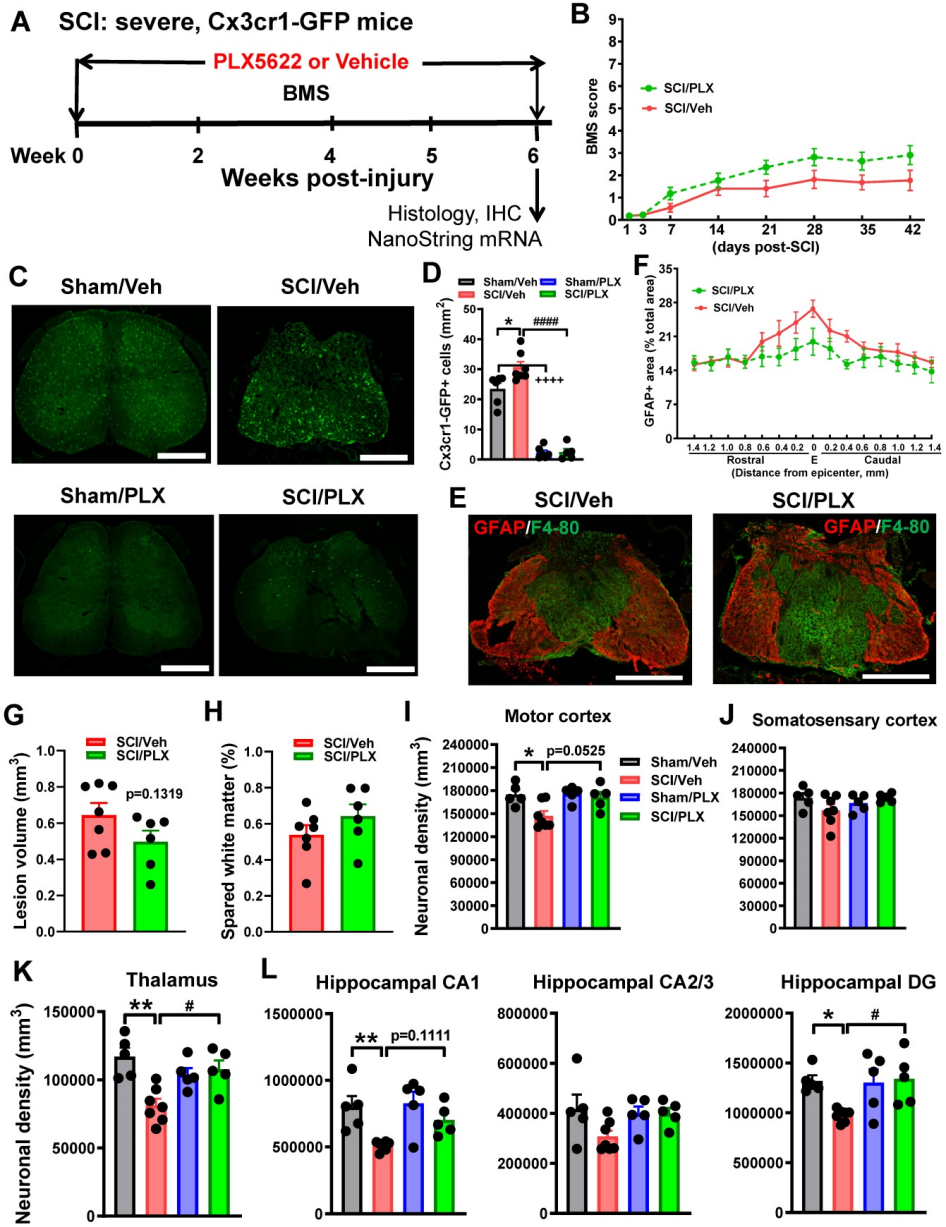
** Depletion of microglia after SCI reduces neurodegeneration in the brain sub-regions.** (**A**) Experimental timeline: adult male CX3CR1-GFP mice underwent severe contusion injury at T10. Right after the surgery, PLX5622 (1200 ppm) or Vehicle chow was fed to the mice for the remainder of the study. BMS was recorded on days 1, 3, and weekly up to 6 weeks post-injury. At the 6 weeks post-injury, spinal cord tissue and brain samples were collected for IHC and histological analysis (Stereology). A subset of mice was sacrificed at 6 weeks post-injury and injured spinal cord samples were collected for Nanostring analysis. (**B**) BMS. No significant differences were found in hindlimb locomotor function on the BMS. n = 11 mice/group (2-way ANOVA with repeated measurements following Bonferroni's multiple comparisons test). (**C**) IHC representative images for GFP^+^ cells at 0.4 mm caudal to the epicenter. Scale bar = 500 µm. (**D**) IHC GFP^+^ cell counts [n = 6 (Sham/Veh), 7 (SCI/Veh), 6 (Sham/PLX), and 5 (SCI/PLX)]. *p < 0.05, ++++*p <* 0.0001, vs. Sham/Veh group. ####*p <* 0.0001, vs. SCI/Veh group. 2-way ANOVA following Tukey's multiple comparisons test. (**E,F**) Representative images (E) of the lesion epicenter from mice on Vehicle diet and PLX5622 diet at 6 weeks immunostained for GFAP (red) and F4/80 (blue). Scale bar =500 µm. Quantification of GFAP+ area (% of total section area) from 1.4 mm rostral or caudal to the lesion epicenter (E). N = 5 mice/group. 2-way ANOVA following Sidak's multiple comparisons test. (**G,H**) Lesion volume (Unpaired t test) and spared whiter matter area (Mann Whitney test) by Stereological analysis. [n = 7 (SCI/Veh) and 6 (SCI/PLX)]. (**I-L**) Neuronal density was assessed by Stereological analysis in motor cortex (M1 and M2), somatosensory cortex (S1 and S2), thalamus (VPL, VPM, PO, and anterior nucleus), hippocampal CA1, CA2/3, and DG regions. Approximate positions of relevant anatomical structures were followed by mouse brain atlas overlay (http://www.mbl.org/atlas170/atlas170_frame.html). [n = 5 (Sham/Veh), 7 (SCI/Veh), 5 (Sham/PLX), and 5 (SCI/PLX)]. *p < 0.05, **p < 0.01, vs. Sham/Veh group. #*p <* 0.05, vs. SCI/Veh group. 2-way ANOVA following Tukey's multiple comparisons test.

To determine whether the effects of microglial depletion by PLX5622 on locomotor function were dependent on injury-severity, Cx3cr1-GFP male mice were subjected to severe contusion injury at T10. When after injury, mice were fed the PLX5622 diet or vehicle diet for the duration of the study (**Figure 5A**). Analysis of the hindlimb locomotor function using BMS did not reveal differences between drug- or vehicle-treated groups (**Figure 5B**). Cx3cr1 is the chemoattractant cytokine Cx3cl1 receptor and Cx3cr1-GFP mice express the enhanced GFP in microglia, monocytes, dendritic cells, and NK cells. IHC was performed on processed spinal cord at 6 weeks post-injury. When compared to control levels in vehicle-treated sham mice, GFP^+^ cell counts revealed a 91% reduction with 6 weeks of PLX treatment (**Figure 5C**). Whereas SCI/Veh mice showed significant increases of GFP^+^ cells compared with Sham/Veh group, PLX treatment led to almost complete microglial elimination (>92% reduction) as demonstrated by loss of cx3cr1-GFP^+^ cells by immunofluorescence (GFP^+^) analyses (**Figure 5D**). GFAP^+^ area analysis showed that PLX5622 treatment did not significantly alter SCI-induced changes in GFAP+ area (**Figure 5E-F**). Consistent with BMS data, histopathological analysis by unbiased stereology at 6 weeks post-injury indicated that there was no difference in lesion volume or spared white matter area between SCI/Veh and SCI/PLX groups (**Figure 5G-H**).

To investigate the effect of systemic depletion of microglia after SCI on brain neuropathology, neuronal density was quantified on processed brain sections stained with cresyl violet at 6 weeks post-injury. Approximate positions of relevant anatomical structures were followed by mouse brain atlas overlay (http://www.mbl.org/atlas170/atlas170_frame.html). Stereological assessment revealed that SCI resulted in significant reduction of neuronal density in motor cortex (M1, M2), thalamus (VPL, VPM, PO, and anterior nucleus), hippocampal CA1 and DG regions compared with Sham/Veh mice (**Figure 5I-L**). Notably, PLX treatment reduced neuronal loss in both the thalamus and DG. There was no difference of neuronal density in subsectors of the somatosensory cortex (S1 and S2) or hippocampal CA2/3 among four groups. A trend of increased neuronal density in motor cortex (*p=*0.0525) and hippocampal CA1 (*p=*0.1111) was shown in the SCI/PLX group without reaching statistical differences. All representative images are indicated in Figure S5.

### Long-term elimination of microglia reduces the transcriptional inflammatory response in the injured spinal cord

**Figure 6:**
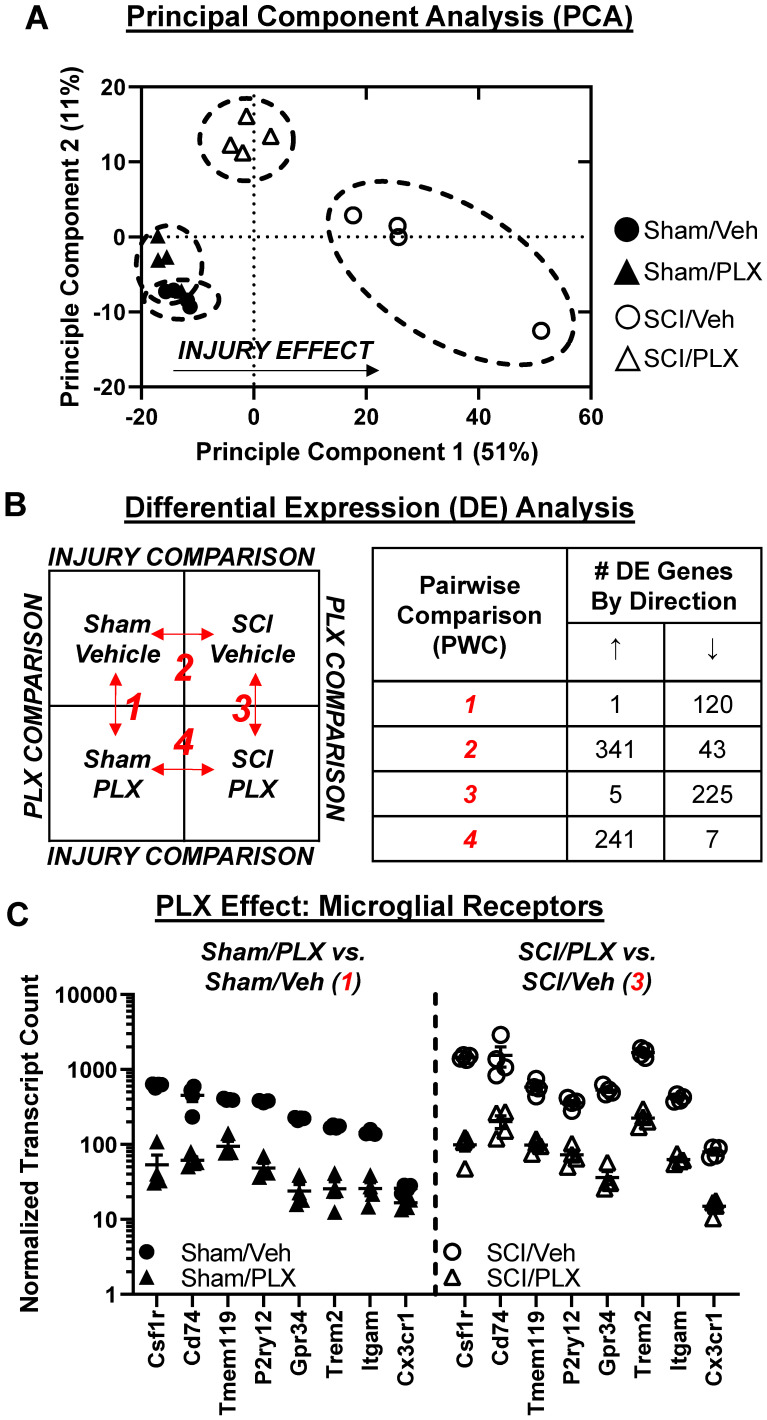
** Long-term depletion of microglia with PLX after SCI significantly alters the transcriptome at the injury site.** A NanoString nCounter® Neuroinflammation panel was used to assess transcriptional changes at the spinal cord injury site at 6 weeks post-injury. (**A**) Principle component analysis (PCA) was performed using all normalized gene counts from the NanoString panel. The four sample groups were Sham/Veh (closed circles), Sham/PLX (closed triangles), SCI/Veh (open circles), and SCI/PLX (open triangles). PCA revealed distinct clustering (dashed ellipses) of the four sample groups across the first two principle components, PC1 and PC2, which accounted for 51% and 11%, respectively, of the total variation across samples. Injury-related effects were captured on PC1, separating the SCI/Veh group on the right from the Sham groups on the left. The SCI/PLX group was near the midline of the axis, indicating a reduction of the injury effect. (**B**) Differential expression (DE) analysis was performed on pairwise group comparisons using NanoStringDiff with a Benjamini-Hochberg false discovery rate correction (adjusted *p*-value < 0.05). Four pairwise comparisons were performed: (1) Sham/PLX vs. Sham/Veh; (2) SCI/Veh vs. Sham/Veh; (3) SCI/PLX vs. SCI/Veh; and (4) SCI/PLX vs. Sham/PLX. DE expressed genes in Injury Comparisons 2 and 4 had predominantly increased fold-change while PLX Comparisons 1 and 3 had predominantly decreased fold-change. (**C**) Plot of transcript counts for well-established microglial receptor markers (Csf1r, Cd74, Tmem119, P2ry12, Gpr34, Trem2, Itgam, Cx3cr1), indicating an effective depletion of microglia by PLX. All receptors had decreased gene expression in both comparisons (adjusted *p*-value < 0.05), except for Cx3cr1 in PLX Comparison 1 due to low baseline counts. n = 4 mice/group. Data presented as individual data points with mean ± S.E.M. Sham/Veh (closed circles), Sham/PLX (closed triangles), SCI/Veh (open circles), and SCI/PLX (open triangles).

To determine whether behavioral improvements with PLX5622 treatment after SCI were associated with transcriptional changes, we evaluated the injury site with NanoString's nCounter® technology at 6 weeks (**Figure 5A**). Over 700 genes were analyzed across three fundamental themes: Immunity and Inflammation (6 pathways); Neurobiology and Neuropathology (13 pathways); and Metabolism and Stress (4 pathways). Principal component analysis (PCA) of all normalized gene counts revealed a distinct separation of samples into individual groups across the first two principle components (**Figure 6A**). The first principle component (PC1) accounted for the majority of the variation (51%) across samples and separated the groups well by injury. Notably, the SCI/PLX group was closer to the Sham groups than the SCI/Veh group along PC1, indicating a reduction of the injury effect at a global transcriptional level.

Differential expression analysis using NanoStringDiff demonstrated a robust number of differentially expressed (DE) genes between groups (adjusted *p*-value < 0.05). Four pairwise comparisons were performed: (1) Sham/PLX vs. Sham/Veh - PLX Comparison 1; (2) SCI/Veh vs. Sham/Veh - Injury Comparison 2; (3) SCI/PLX vs. SCI/Veh - PLX Comparison 3; and (4) SCI/PLX vs. Sham/PLX - Injury Comparison 4 (**Figure 6B**). SCI resulted in primarily increased expression of genes in both Veh-treated and PLX-treated group comparisons whereas PLX treatment resulted in decreased expression of genes in both Sham and SCI group comparisons (**Figure 6B**). PLX treatment resulted in a significant reduction of gene transcript counts for well-established microglia receptor markers, indicating an effective elimination of the majority of microglia (**Figure 6C**). All microglial receptors (Csf1r, Cd74, Tmem119, P2ry12, Gpr34, Trem2, Itgam) were reduced by at least 75% with PLX in both Sham and SCI group comparisons except for Cx3cr1, which had a low mean baseline count in Sham/Veh animals (**Figure 6C**).

**Figure 7:**
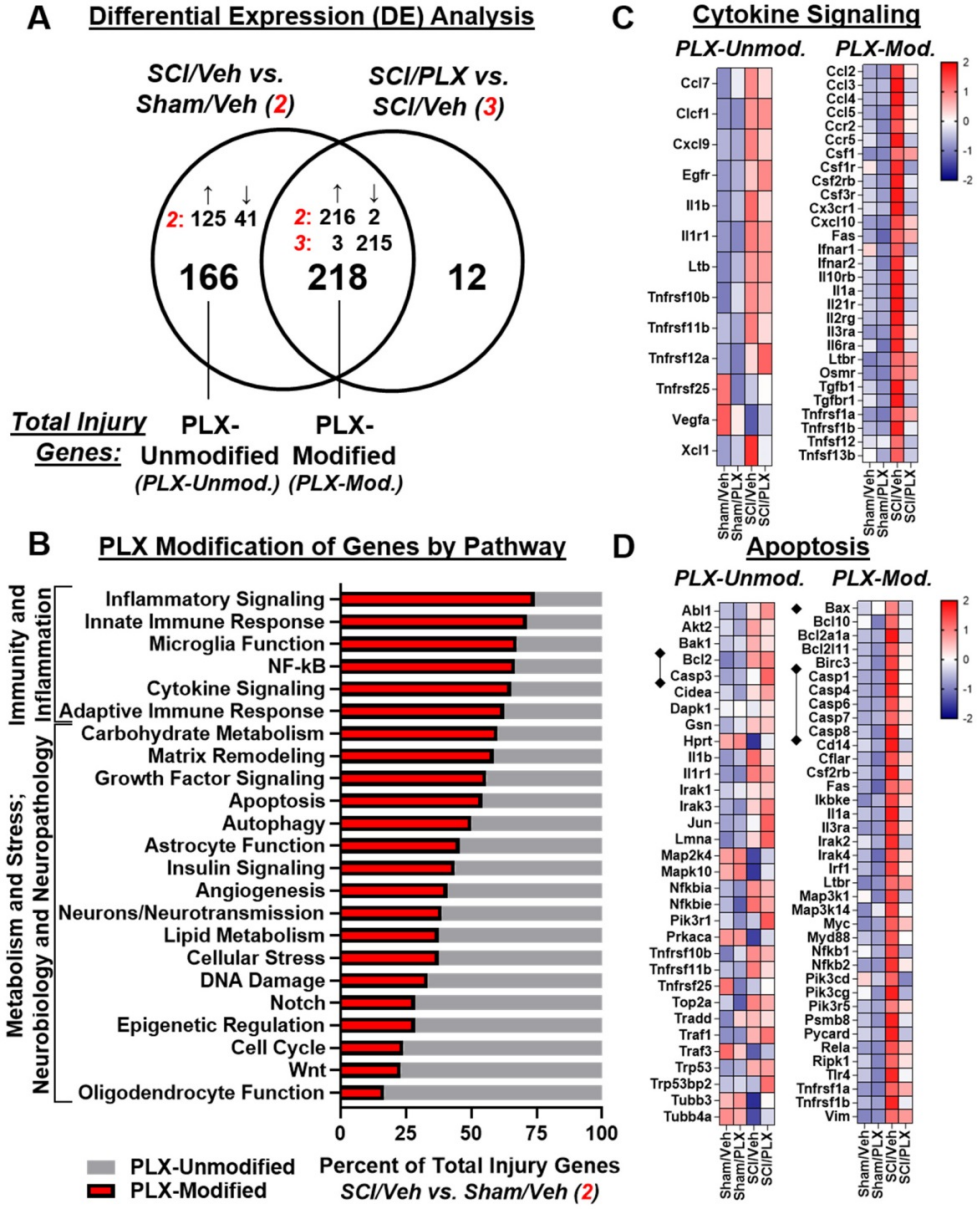
** Long-term depletion of microglia after SCI significantly reduces expression of genes related to inflammation and apoptosis in the spinal cord.** (**A**) Venn diagram demonstrates the separation of total injury genes (*i.e.*, those genes differentially expressed in SCI/Veh vs. Sham/Veh - Injury Comparison 2) into those modified by PLX (PLX-Modified) or not modified by PLX (PLX-Unmodified) based on membership in the gene list of PLX Comparison 3 (SCI/Veh vs. SCI/PLX). 57% of total injury genes (218 of 384) were PLX-modified, and all of these genes except one (217 of 218) had an attenuation of the original injury effect. The remaining 43% of injury genes (166 out of 384) were not modified by PLX. (**B**) Graph illustrating the percent distribution of total injury genes by pathway annotation (and theme) that are PLX-Modified or PLX-Unmodified. The top 6 pathways with the greatest proportion of PLX-modification were all within the theme of Immunity and Inflammation. (**C**) Heatmap of genes related to cytokine signaling that are differentially expressed (DE) by injury and PLX-Unmodified (left) or PLX-Modified (right). Color coding was based on z-score scaling. (**D**) Heatmap of genes related to apoptosis that are DE by injury and PLX-Unmodified (left) or PLX-Modified (right). Color coding was based on z-score scaling. The majority of caspases have increased gene expression with injury that is decreased with PLX with the exception of Casp3. Expression for the pro-apoptotic mitochondrial permeabilizing protein, Bax, is increased with injury and decreased with PLX treatment while PLX treatment does not affect expression of the associated anti-apoptotic gene, Bcl2.

We then compared the overlap of DE gene lists between comparisons SCI/Veh vs. Sham/Veh and SCI/PLX vs. SCI/Veh to determine which injury genes were modified by PLX treatment. Out of the 384 total injury genes, 218 (57%) were modified by PLX while 166 (43%) were not modified by PLX (**Figure 7A**). All PLX-Modified injury genes had an attenuation of the magnitude of the original injury effect (*i.e.*, genes that increased by injury were decreased by PLX and vice versa) except for just a single gene, Mfge8, which further increased in SCI/PLX relative to SCI/Veh (**Figure 7A**). One of the functions of lactadherin, the protein encoded by Mfge8, is to aid in the phagocytic clearance of apoptotic cells through binding to phosphatidylserine exposed on the surface of these cells. Thus, the increased expression in the SCI/PLX group may represent compensatory production from non-microglial cells or from the small percentage of residual microglia.

PLX treatment had a broad effect on injury genes across multiple biological themes. Consistent with a critical role for microglia in neuroinflammation, the top 6 pathways with the greatest proportion of injury genes that were PLX-Modified were all related to Immunity and Inflammation: Inflammatory Signaling, Innate Immune Response, Microglia Function, NF-κB, Cytokine Signaling, and Adaptive Immune Response (**Figure 7B**). PLX treatment reduced gene expression levels of a number of injury-induced chemokines, cytokines, and associated receptors (**Figure 7C**). In addition to a reduction in the inflammatory response transcriptionally, nearly 50% of injury genes related to the processes of apoptosis (**Figure 7D**) and autophagy (**Figure S6A**) were modified by PLX and critically impact neuronal cell loss after SCI. Importantly, there was decreased gene expression of many caspases (Casp1, Casp4, Casp6, Casp7, Casp8) by PLX that are upregulated after injury, although not Casp3 (**Figure 7D**). PLX also reduced expression of the pro-apoptotic gene, Bax, whose protein product permeabilizes the mitochondria resulting in the release of cytochrome c as part of the intrinsic apoptotic pathway; the associated anti-apoptotic gene, Bcl2, had increased transcript counts with injury but was not further modified by PLX treatment (**Figure 7D**). A significant part of the transcriptional profile associated with astrocyte function, growth factor signaling, and matrix remodeling was also modified with PLX treatment, which may also contribute to improved repair and recovery (**Figure S6B-C**).

**Figure 8:**
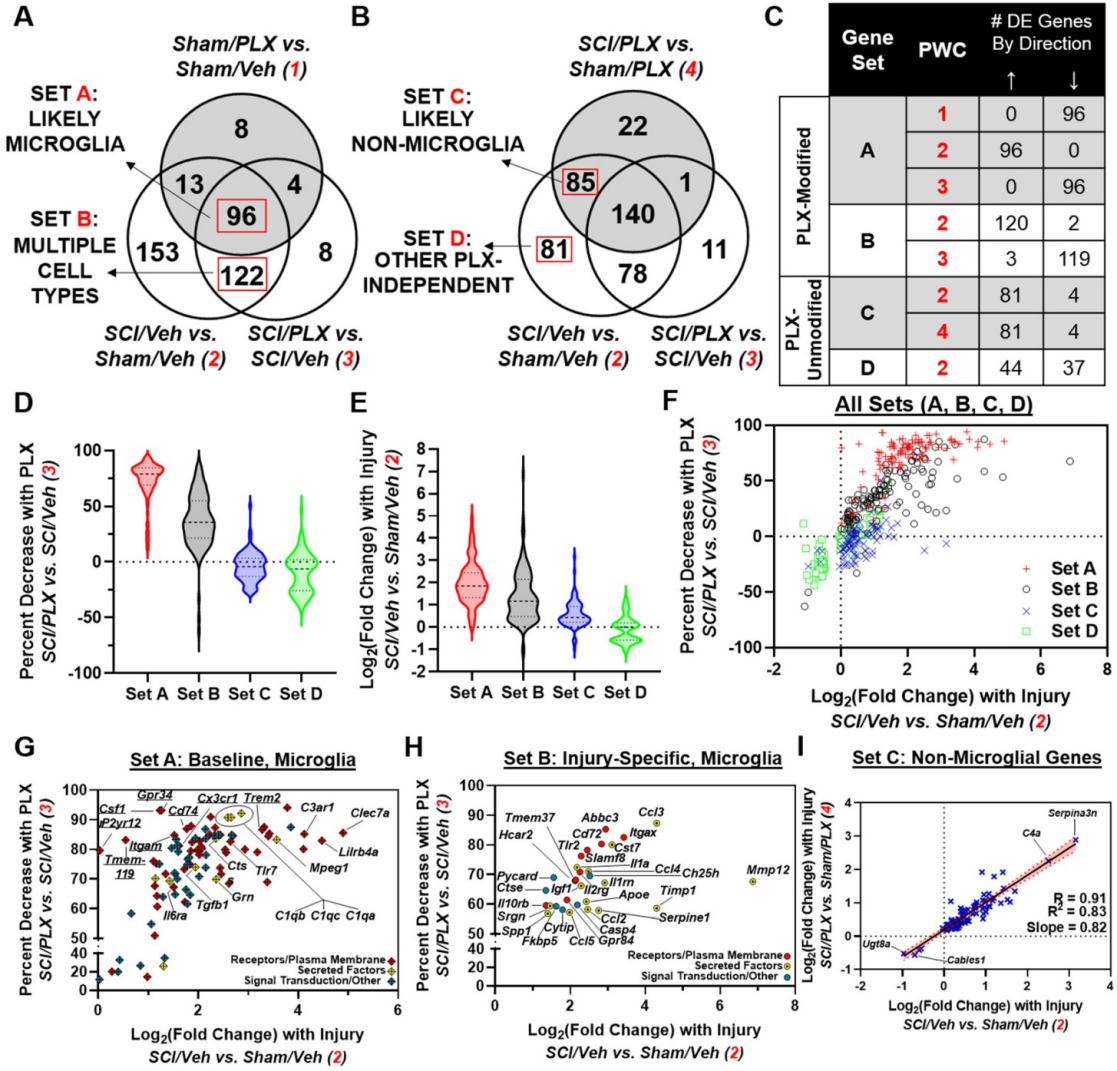
** Distinct transcriptional components of the inflammatory response are driven by microglia after SCI.** (**A**) Venn diagram showing the overlap of three different comparisons: *SCI/Veh vs. Sham/Veh* (*Injury Comparison 2*), *SCI/PLX vs. SCI/Veh (PLX Comparison 3*), and* Sham/PLX vs. Sham/Veh* (*PLX Comparison 1*). PLX-Modified injury genes are divided into two sets, A and B, based on their membership in *PLX Comparison 1*. Based on these comparisons, Set A likely contains a majority of microglial genes while Set B contains a mixture of genes from microglia (more injury-related) and other cell types. (**B**) Venn diagram showing the overlap of three different comparisons: *SCI/Veh vs. Sham/Veh* (*Injury Comparison 2*), *SCI/PLX vs. SCI/Veh (PLX Comparison 3*), and* SCI/PLX vs. Sham/PLX* (*PLX Comparison 4*). PLX-Unmodified injury genes are divided into two sets, C and D, based on their membership in *PLX Comparison 4*. Based on these comparisons, Set C likely contains non-microglial genes that respond to injury irrespective of the presence of microglia while Set D also includes other non-microglial genes. (**C**) Table showing the number and direction of differentially expressed genes within a Set for each pairwise comparison (PWC) that defines the membership in that Set. All genes in Set A were increased with injury (*Injury Comparison 2*), and decreased with PLX in both Sham and SCI animals (*PLX Comparisons 1 and 3*, respectively.) All the genes in Set B (120 upregulated and 2 downregulated with injury) except for one had the opposite direction of change between *Injury Comparison 2* and *PLX Comparison 3*. All 85 genes in Set C had the same direction of change in both *Injury Comparison 2* and *Injury Comparison 4*. Genes in Set D were only found in *Injury Comparison 2* (44 upregulated; 37 downregulated). (**D**) Violin plots showing the distribution of genes in each Set based on percent decrease with PLX after SCI. Set A contained the greatest percent reduction (median = 79%; first quartile = 69%; third quartile = 85%) followed by Set B (median = 35.7%; first quartile = 21.47%; third quartile = 55%), consistent with the fact that Set A contains a majority of microglial genes, while Set B contains only some microglial genes. Median values for each set are marked with a dashed line while the first and third quartiles are marked with dotted lines in the violin plots. (**E**) Violin plots showing the distribution of genes in each Set based on log_2_(Fold Change) with *SCI/Veh vs. Sham/Veh*. Sets A and B had the highest median log_2_(Fold Change) with values of 1.85 (first quartile = 2.45; third quartile = 1.33) and 1.17 (first quartile = 2.15; third quartile = 0.48), respectively. Median values for each set are marked with a dashed line while the first and third quartiles are marked with dotted lines in the violin plots. (**F**) Plot of each gene by log_2_(Fold Change) in *SCI/Veh vs. Sham/Veh* and percent decrease with PLX in *SCI/PLX vs. SCI/Veh*. Genes were plotted by Set membership: Set A, red “+” signs; Set B, black open circles; Set C, blue “x” signs; Set D, green open squares. (**G**) Plot of genes only in Set A by log_2_(Fold Change) in *SCI Veh vs. Sham Veh* and percent decrease with PLX in *SCI/PLX vs. SCI/Veh*. A large majority of these genes (54 out of 96) encode for receptors or other plasma membrane components (red diamond “+” signs) while a minority (10 out of 96) encode for secreted factors (yellow diamond “+” signs). The remaining genes (32 out of 96) encode for signal transduction or other proteins (blue diamond “+” signs.) Genes of particular interest noted in the body of the text are labeled. (**H**) Plot of a subset of 30 genes in Set B by log_2_(Fold Change) in *SCI/Veh vs. Sham/Veh* and percent decrease with PLX in *SCI/PLX vs. SCI/Veh*. These genes display similar log_2_(FC) and percent decrease with PLX after SCI to genes in Set A, suggesting that these may be microglia genes that are specifically induced after injury. A significant number of these candidate genes encode for secreted molecules (14 out of 30; yellow circles with center dot) followed by receptors/plasma membrane components (10 out of 30; red circles). The remaining minority of genes (6 out of 30) encode for signal transduction or other proteins (blue circles). All genes are labeled on the plot. (**I**) Plot of the log_2_(FC) in both *Injury Comparisons 2* and *4* for genes in Set C. Linear regression analysis shows a strong correlation (R = 0.91; R^2^ = 0.83) with slope close to 1 (slope = 0.82), supporting the notion that these genes are likely being modified by injury in non-microglial cells and respond independent of microglia. The red dotted lines and shaded area represent the upper and lower 95% confidence intervals for the regression analysis. Genes of particular interest noted in the body of the text are labeled.

To further delineate which injury genes may be more specific to the microglial response, we considered the overlap of total injury genes partitioned by PLX modification (Venn diagram in **Figure 7A**) with DE genes in the Sham/PLX vs. Sham/Veh comparison (**Figure 8A**) or SCI/PLX vs. Sham/PLX comparison (**Figure 8B**). We took advantage of the fact that long-term depletion of microglia with PLX in Sham animals identified over 100 genes that had decreased expression (**Figure 6B**), indicating their presence in microglia at baseline. We then divided PLX-Modified injury genes into two groups, Sets A and B, based on their membership in Sham/PLX vs. Sham/Veh (**Figure 8A**).

Set A contained 96 genes that were most likely expressed in microglia. These genes increased with injury and decreased with PLX in both Sham and SCI comparisons (**Figure 8C**). Genes in Set A had the highest percent reduction by PLX after SCI (median = 79%; first quartile = 69%; third quartile = 85%) and the highest fold-change increase with injury (median = 1.85; first quartile = 1.33; third quartile = 2.45) (**Figure 8D-F**). A large majority of the genes in Set A (54 out of 96) were plasma membrane components associated with microglial activation including Toll-like receptors (*e.g.*, Tlr7); immunoglobulin and Ig-like receptors (*e.g.*, Lilrb4a); and receptors for complement (*e.g.*, C3ar1), lectin (*e.g.*, Clec7a) and cytokines (*e.g.*, Il6ra) (**Figure 8G**). All microglial receptor markers identified in **Figure 6C** had increased gene expression with injury, though P2yr12 and Tmem119 had log2(FC)<1. There was also injury-induced upregulation of some secreted molecules (10 out of 96) expressed at baseline in microglia such as Mpeg1, Ctss, Grn, Tgfb1 and complement factors C1qa, C1qb, and C1qc (**Figure 8G**).

Set B contained PLX-Modified injury genes that were not altered in Sham/PLX vs. Sham/Veh. We reasoned that Set B contained two categories of genes: (1) genes expressed in non-microglia cells that are affected secondarily by PLX treatment; (2) a subset of microglial genes that are induced specifically after injury and not expressed in microglia at baseline. As shown in **Figure 8F**, some Set B genes clustered well with Set A genes, exhibiting similar characteristics in terms of injury fold-change and percent PLX reduction. These potential injury-specific microglia genes included a greater enrichment of secreted molecules (14 out of 30) including chemokines/cytokines (Ccl2, Ccl3, Ccl4, Ccl5, Il1a, Igf1, Spp1) and extracellular-matrix modifying factors (Mmp12, Timp1, Serpine1, Apoe) (**Figure 8H**). SCI also induced gene expression of certain receptors in microglia, including Tlr2 and Itgax (encoding CD11c), that are only minimally expressed at baseline (**Figure 8H**).

In addition to the identification of microglial genes, our analysis also identified injury genes that respond independent of microglia. By including the SCI/PLX vs. Sham/PLX comparison, we divided injury genes that are not modified by PLX into two groups, Sets C and D (**Figure 8B**). Set C included genes (81 upregulated; 4 downregulated) that were modified with injury in both Veh-treated and PLX-treated mice (**Figure 8C**). Linear regression analysis of the injury fold-change in each comparison for these genes demonstrated a strong correlation (R=0.91, R^2^=0.83) with a slope close to 1, indicating that they are modified by injury to a similar magnitude with or without microglia (**Figure 8I**). Within Set C, the top two genes with the greatest increase with injury were Serpina3n and C4a, both associated with astrocyte function, while the top two genes with the greatest decrease with injury were Ugt8a and Cables1 (**Figure 8I**).

Finally, we performed a network analysis to investigate the biological relationships between genes within our set classification scheme. We used the STRING interaction database to create a map based on known protein-protein interactions (PPI). There were a strong number of connections between microglial genes defined in Set A (**Figure S7A**). Additionally, there was a high number of connections between our potential injury-specific microglia genes in Set B; these included signaling molecules related to chemokines/cytokines (Ccl2, Ccl3, Ccl5, Il1a), type 1 interferons (Irf1, Ifnar1), inflammasome (Casp1), NFκB (Myd88, Rela/Relb, Nfkb1/Nfkb2) and Toll-like receptor 2 (Tlr2) **(Figure S7A**, inset, red asterisks). When all injury genes were placed into a single PPI network map, the degree of connectivity - defined for a single protein as the number of direct protein interactions - was highest for genes in Sets A and B (**Figure S7B**). We then created a subnetwork consisting of genes with degree greater than 50 in Sets A and B and displayed their relationships in a pathway diagram in **Figure S7C**. Collectively, our transcriptional analysis through a combined bioinformatics and biological network approach highlight a critical function of microglia in neuroinflammation after SCI.

**Figure 9:**
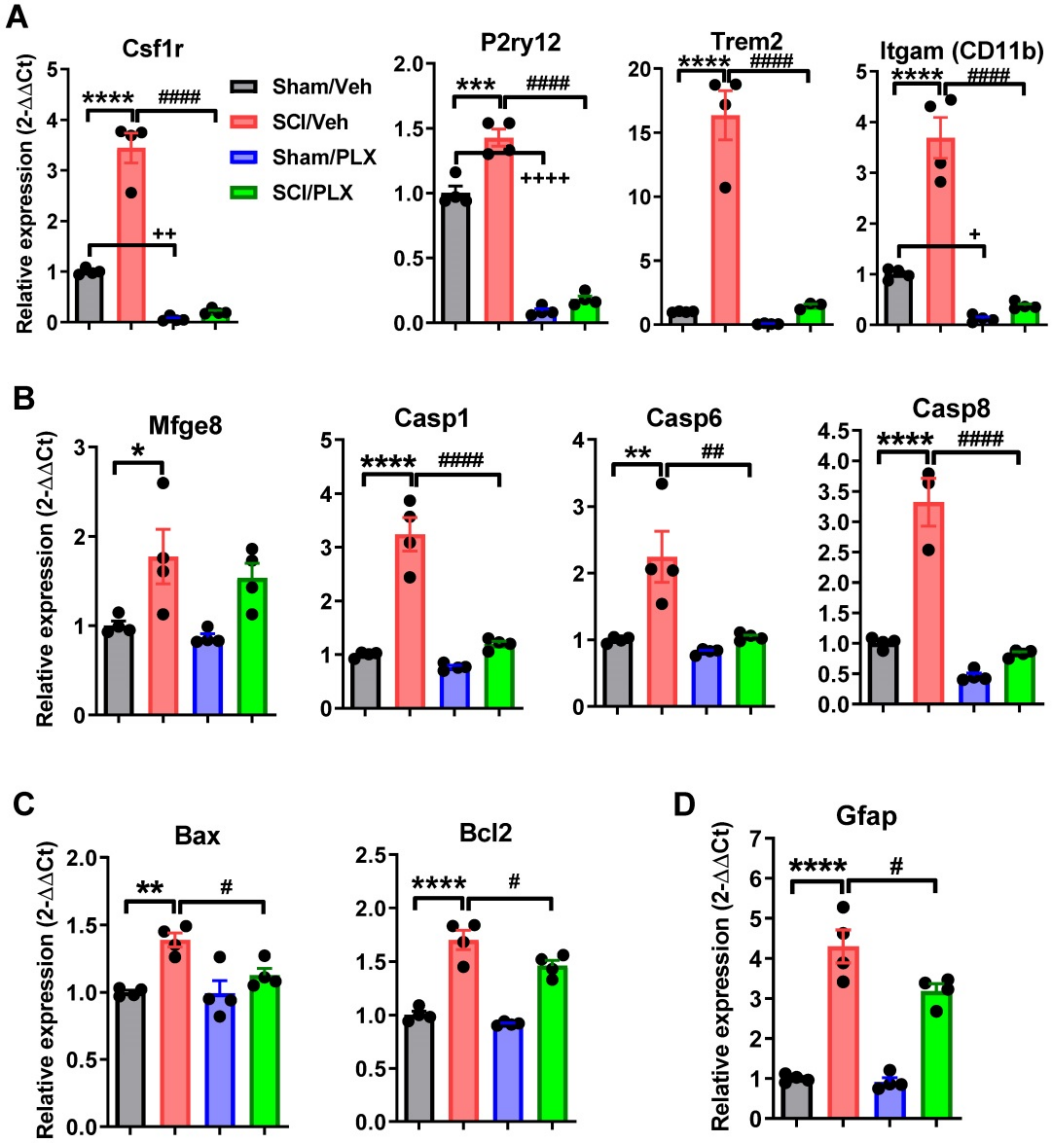
** The effects of microglial depletion on the expression levels of microglial receptors and biomarker genes of neuronal apoptosis and astrocytes in the spinal cord at 6 weeks post-injury.** (**A**) Microglial receptors including Csf1r, P2ry12, Trem2, and Itgam (CD11b) were significantly upregulated following injury and reduced with PLX treatment in both Sham and SCI groups. (**B-C**) The markers related to the processes of neuronal apoptosis and autophagy. (**D**) Astrocyte marker Gfap. All data are presented as independent data points. N = 4 mice/group. * or +*p <* 0.05, ** or ++*p <* 0.01, ***p < 0.001, **** or ++++*p <* 0.0001, vs. Sham/Veh group; #*p <* 0.05, ##*p <* 0.01, ####*p <* 0.0001 vs. SCI/WT. Two-way ANOVA following Tukey's multiple comparisons test.

To further confirm these microglial depletion-related signaling pathways in injured spinal cord, we used qPCR to examine the gene expression levels of microglial receptors and biomarkers of neuronal apoptosis and astrocytes. As expected, microglial receptors including Csf1r, P2ry12, Trem2, and Itgam (CD11b) were significantly upregulated following injury (*p <* 0.0001 vs Sham/Veh group, **Figure 9A**), and reduced with PLX treatment in both Sham and SCI groups. Next, we determined the expression level of the genes related to the processes of neuronal apoptosis and autophagy including Mfge8, Casp1, 6, 8, Bax, and Bcl2. Consistent with our previous results, SCI caused a significant increase in all neuronal apoptotic markers (**Figure 9B-C**). Notably, PLX treatment in SCI mice robustly reduced the expression of these genes relative to SCI/Veh group. Finally, there was significantly decreased expression of the astrocyte marker Gfap by PLX that was upregulated after injury (**Figure 9D**).

### SCI alters neuronal gene expression in the cortex while long-term PLX treatment modulates both microglial and astrocyte-related inflammation

**Figure 10:**
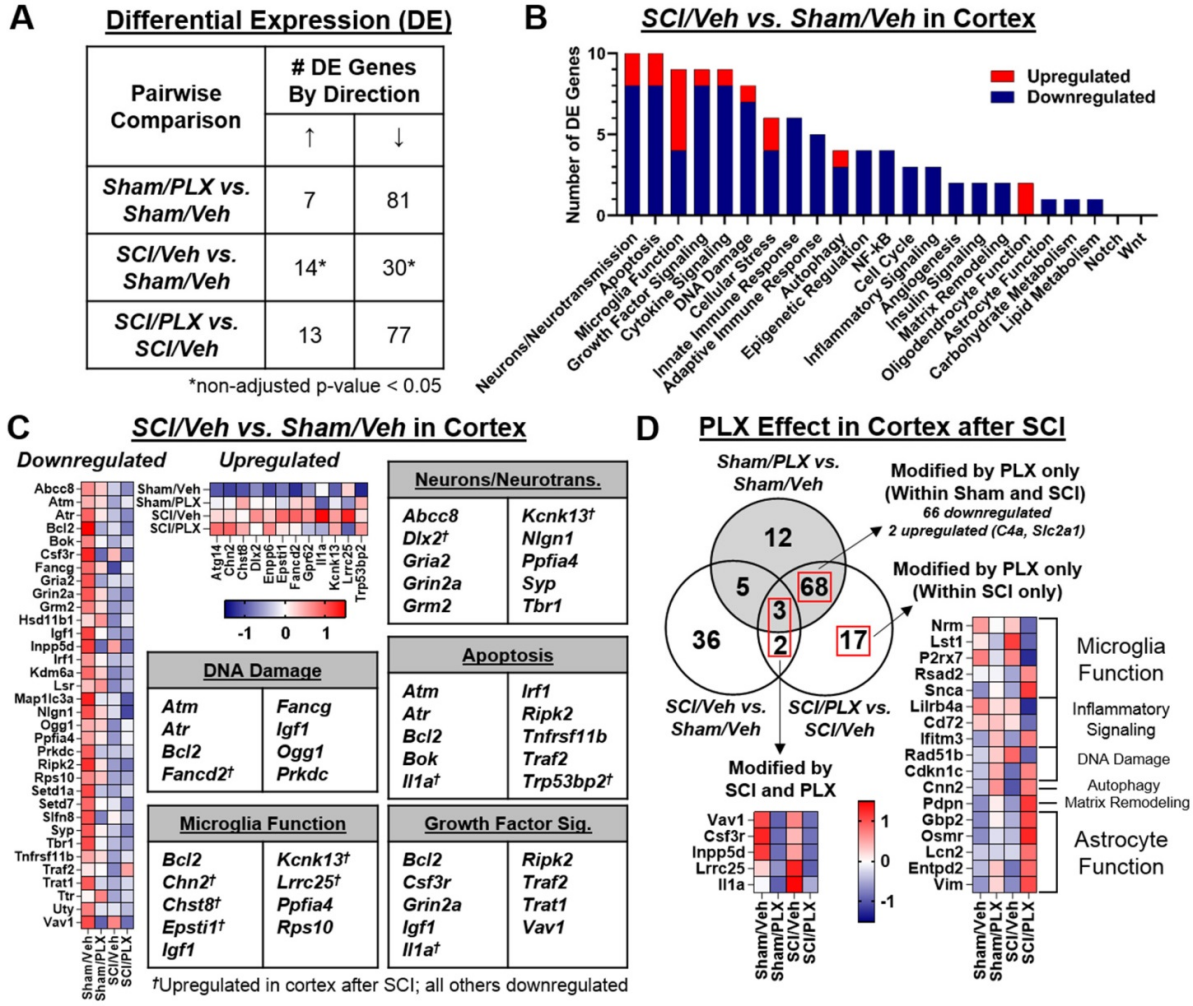
** SCI decreases transcriptional activity in the cortex related to neuronal synaptic function while long-term PLX depletion in the context of SCI increases astrocyte-related gene expression.** (**A**) Table showing the number of differential expressed (DE) genes in the somatosensory cortex by pairwise comparison using NanoStringDiff. N = 4 mice/group. The three comparisons were as follows: (*1) Sham/PLX vs. Sham/Veh; (2) SCI/Veh vs. Sham/Veh;* and *(3) SCI/PLX vs. SCI/Veh*. About two-thirds of DE genes in the cortex after SCI were downregulated (*SCI/Veh vs. Sham/Veh*). PLX treatment in both Sham and SCI animals resulted in largely decreased expression of genes. (**B**) Distribution of DE genes in the cortex for the *SCI/Veh vs. Sham/Veh* comparison by pathway annotation. The neurons/neurotransmission and apoptosis pathways had the highest number of DE genes, with eight out of ten decreased in each category. Microglia function had the next highest number of DE genes as well as the highest number of genes with increased expression of any pathway (five). (**C**) Heatmap of DE genes in the cortex after SCI that are either downregulated (left; 30 genes) or upregulated (right; 14 genes). Color coding was based on z-score scaling. Individual tables show lists of DE genes by specific pathway. The “†” sign denotes genes that are upregulated. (**D**) Venn diagram showing the overlap of the DE gene lists for the three pairwise comparison. The central overlap area identifies five genes that are both modified by injury as well as PLX treatment after SCI and shown in the corresponding heatmap (color coding based on z-score scaling.) Two genes, *Il1a* and *Lrrc25*, were increased with injury in the cortex and decreased with PLX treatment. 68 genes were modified by PLX only in both Sham and SCI animals, and only two of these genes had increased expression (*C4a*, *Slc2a1*). A group of 17 genes were modified by PLX only in SCI animals. 11 out of 17 genes were increased in these animals relative to the SCI Veh group, and five of these upregulated genes were specifically associated with astrocyte function.

To determine whether there were also transcriptional changes in the brain after SCI that could be modified by PLX treatment, we analyzed the dissected cortex (motor cortex and somatosensory cortical area) using the same nCounter® Neuroinflammation panel. We found that 30 out of a total 44 differentially expressed (DE) genes in the cortex were decreased after SCI (non-adjusted *p*-value < 0.05; **Figure 10A**). Similar to the spinal cord, PLX treatment in Sham and SCI animals resulted in largely decreased expression of genes (adjusted *p*-value < 0.05; **Figure 10A**). Altered gene expression in the cortex after SCI was strongly related to neurons and neurotransmission, apoptosis, and microglia function (**Figure 10B**). We found decreased gene expression of a number of synaptic proteins including ionotropic and metabotropic glutamate receptors (*i.e.*, Gria2, encoding an AMPA receptor subunit; Grin2a, encoding a NMDA receptor subunit; and Grm2, encoding mGlur2) as well as Nlgn1 and Syp, which are related to synaptic organization and synaptic vesicle function, respectively (**Figure 10C**). Genes related to apoptosis, DNA damage/cell cycle (*e.g.* Atm, Atr, Prkdc) and growth factor signaling (*e.g.* Igf1) were primarily decreased in the cortex, including the important anti-apoptotic gene, Bcl2 (**Figure 10C**). On the other hand, five of the 14 genes that increased in the cortex after SCI were related to microglia function (*i.e.*, Chn2, Chst8, Epsti1, Kcnk13, Lrrc25) while two other increased genes were associated specifically with pro-apoptotic activity: Il1a, a pleiotropic cytokine gene, and Trp53bp2, a gene encoding a p53 interacting protein (**Figure 10C**). Finally, to determine whether PLX treatment reversed any SCI-induced transcriptional changes in the cortex, we compared the overlap of DE gene lists in comparisons for SCI/Veh vs. Sham/Veh, SCI/PLX vs. SCI/Veh, and Sham/PLX vs. Sham/Veh (**Figure 10D**). While only a few injury-induced genes in the cortex were modified by PLX, we identified Il1a and Lrrc25 as two genes that increased with injury and decreased with PLX treatment, suggesting that microglia may be the source of their expression in the cortex after SCI. We also found a high proportion of genes (11 out of 17) that were increased specifically in the SCI/PLX vs. SCI/Veh comparison (**Figure 10C**). Five of these increased genes are associated with astrocyte function - Gbp2, Osmr, Lcn2, Entpd2, and Vim - and all of these genes were in fact decreased at the injury site (**Figure S6B**). Two other genes were increased with PLX treatment in both Sham and SCI animals: C4a, found primarily in astrocytes, and Slc2a1, which encodes the major glucose transporter, GLUT1, in the blood-brain barrier (**Figure 10C**). Compared to the spinal cord (**Figure 6B**), PLX treatment in the cortex resulted in a higher number of genes with increased expression in both Sham and SCI animals that are likely being expressed in non-microglial cells in response to microglial depletion. Overall, these data support a depression of activity related to neuronal and synaptic function transcriptionally in the motor and somatosensory cortex after SCI, an area directly connected to the injury site, with an associated increase in certain transcriptional activity in microglia. PLX treatment reduced injury-induced expression of Il1a and Lrrc25, while also increasing the expression of a few astrocyte-related genes.

**Figure 11:**
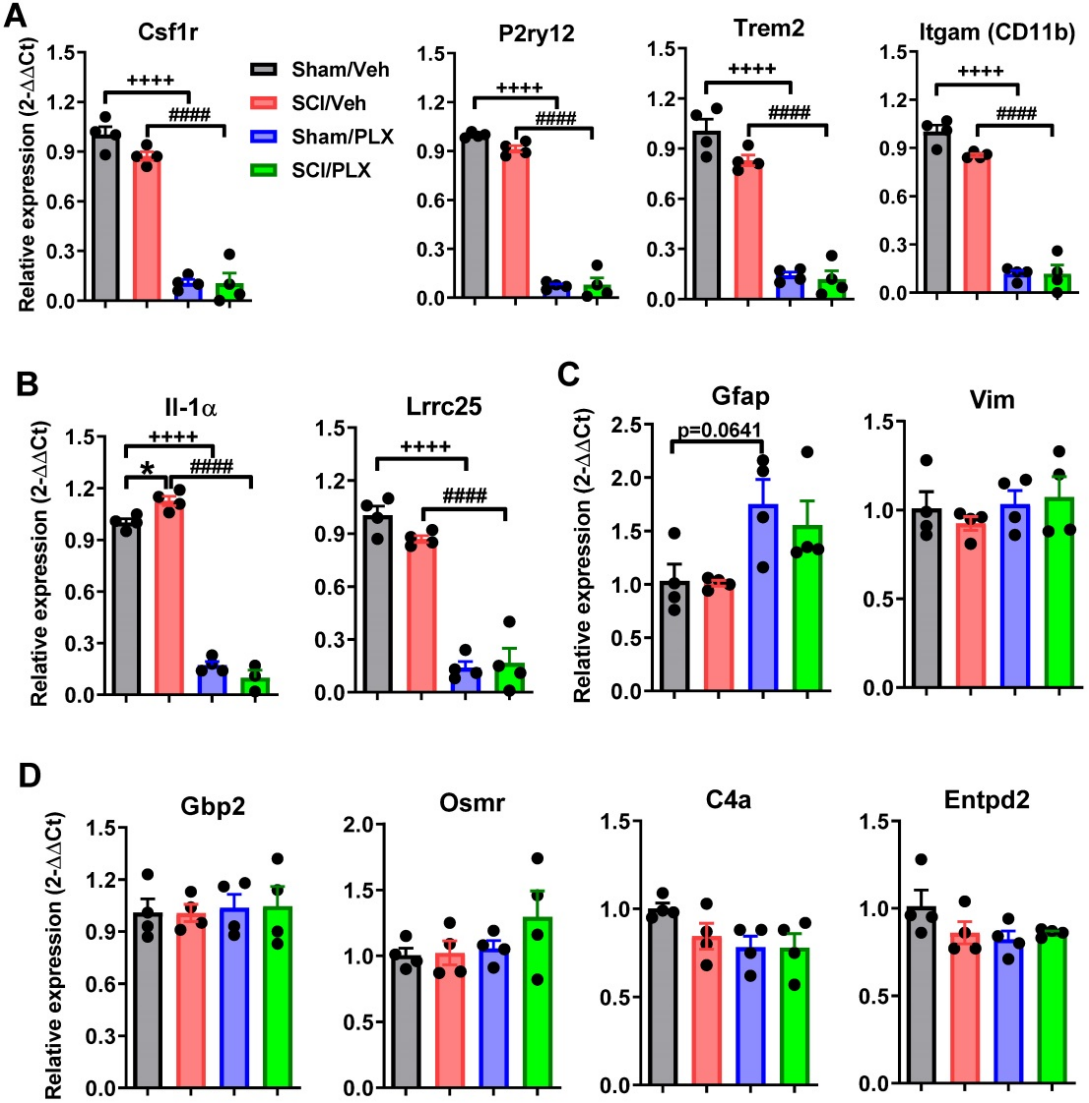
** qPCR analysis in RNA isolated from the cerebral cortex.** (**A**) PLX treatment caused a significant reduction of microglial receptors (Csf1r, P2ry12, Trem2, and Itgam) in both Sham and SCI groups. (**B**) Inflammatory cytokine IL-1α and Lrrc25 expression. (**C-D**) Astrocytes marker Gfap and the genes that are associated with astrocyte function including Vim, Gbp2, Osmr, C4a, and Entpd2 remained unchanged in both SCI and PLX groups. All data are presented as independent data points. N = 4 mice/group. *p < 0.05, ++++*p <* 0.0001, vs. Sham/Veh group; ####*p <* 0.0001 vs. SCI/WT. Two-way ANOVA following Tukey's multiple comparisons test.

qPCR analysis of RNA isolated from the cerebral cortex validated the significant reduction of microglial receptors (Csf1r, P2ry12, Trem2, and Itgam) seen with PLX treatment in both sham and SCI groups (*p <* 0.0001 vs Sham/Veh group, **Figure 11A**). Remarkably, inflammatory cytokine IL-1α expression was significantly induced in the cortex in response to SCI, and however, reduced in PLX-treated animals (**Figure 11B**). Lrrc25, which functions as an inhibitor of NF-κB signaling pathway, remained unchanged with injury, but was significantly decreased with PLX treatment. Unlike at the injured site, the astrocyte marker Gfap was unchanged in the brain after SCI (**Figure 11C**). qPCR analysis also showed no significant alteration of Gfap mRNA level in the cortex by microglial depletion in both sham and SCI groups (**Figure 11C**). Genes associated with astrocyte function including Vim, Gbp2, Osmr, C4a, and Entpd2 remained unchanged in both SCI and PLX groups (**Figure 11C-D**).

## Discussion

Microglial ablation using CSF1R antagonists has been applied to various experimental CNS disease models associated with neuroinflammation 38-42. In the present study, we investigated the effects of microglial depletion with the CSF1R inhibitor PLX5622 later to induction of injury on SCI-mediated brain neuroinflammation and neurological dysfunction. We showed that PLX treatment leads to microglial elimination in both spinal cord and brain, as evidenced by flow cytometry, IHC, and NanoString analysis. Microglial depletion, whether prior or post-injury, also significantly reduced infiltrating monocytes and neutrophils during the acute stages of injury. SCI-induced intraspinal ROS production in these cells and as well as the pro-inflammatory cytokine, TNFα, were downregulated by PLX treatment. Nanostring gene array from the injured spinal cord showed downregulation of a majority of the genes in the inflammatory pathways at 6 weeks after injury with PLX treatment, consistent with the critical function of microglia in chronic neuroinflammation after SCI. Moreover, we demonstrated that SCI altered neuronal gene expression in the cortex, and that long-term PLX treatment modulated both microglial and astrocyte-related inflammation. Treatment also resulted in improved neuronal survival in subregions of the thalamus and DG region of the hippocampus, as well as less fine motor dysfunction, cognitive dysfunction and depressive-like behavior. Together, these data support an important role for neurotoxic microglial activation in regulating posttraumatic neurodegeneration and related neurological impairments after SCI.

CSF1, a macrophage- and monocyte-specific growth factor, is a cytokine that controls the proliferation, differentiation, and function of macrophages 65. The CSF1R gene encoding a tyrosine kinase growth factor receptor for CSF1 mediates most, if not all, of the biological effects of this cytokine. CSF1R is highly expressed by microglia and macrophages in the CNS, and its signaling is required for their proliferation, differentiation, and survival. PLX5622 is a highly potent, brain penetrant and orally active CSF1R inhibitor that can eliminate most adult microglia (>95%) in brain 42. In the present treatment paradigm, we showed that administration of PLX5622 for 3-6 weeks led to more than 90% microglial reduction in the intact thoracic spinal cord, as demonstrated by flow cytometry using CD11bCD45^int^ cell counts and IHC CX3CR1-GFP^+^ cell counts. Seven-day treatment resulted >94% CD11bCD45^int^ cell reduction in the intact brain, but only 70% reduction in spinal cord. The differences between the brain and spinal cord and between different studies may reflect differential the blood velocity profiles, metabolic requirements of cells, microglia cell density, dynamic permeability, or chosen outcome 66, 67.

Intraspinal microglial proliferation and reactivation are among the key secondary injury processes in response to traumatic insults. However, it remains unclear to what extent injury-activated microglia contribute to tissue pathology, as infiltrating leukocytes contribute to secondary pathogenesis and may serve as a key determinant of long-term recovery after SCI 68. Neutrophils and monocytes are the first immune cells to infiltrate the spinal cord after SCI, beginning as early as 12-24 h post-injury and reaching peak levels during the first week 69. Neutrophils can generate oxidative stress, release proteases, recruit further classes of inflammatory cells through chemokine networks, and induce vascular leakage - leading to tissue damage. Infiltrating monocytes also generate inflammatory cytokines and chemokines 70, 71. Consistent with prior reports, increased numbers of monocytes and neutrophils were observed at 2 days post-injury. CD11bCD45^int^ microglia or CX3CR1-GFP^+^ cells, increase by injury, were reduced by long-term treatment of PLX toward levels found in Sham/PLX animals. Notably, continuous PLX treatment significantly attenuated the number of CD45^hi^CD11b^+^ infiltrating myeloid cells compared to that of injured controls; these included both monocytes and neutrophils at 2 and 7 days post-injury. The latter is consistent with the recent report 43 showing robust reduction of LysM-eGFP^+^ myeloid cell recruitment by PLX at 7 days-post injury in transgenic Cx3cr1^creER^:R26-TdT:LysM-eGFP mice. However, Ly6G^+^ neutrophils counted by immunostaining at the lesion center were not altered by PLX at day 1 post-injury 43. This difference may reflect variations in the injury severity or differences in sex or species, as well as treatment paradigm. It is known that one function of leukocytes is to produce ROS, overproduction of which causes oxidative stress and exacerbates tissue damage. PLX treatment reduced both microglial ROS, as well as ROS production by infiltrating monocytes and neutrophils. A large body of evidence indicates that depletion or limitation of inflammatory monocyte infiltration is beneficial to tissue repair 45-49. Although debate remains regarding the role of neutrophils in the injured cord 72, reducing both neutrophil and monocyte recruitment promotes recovery after SCI 49. As the primary immune cell in the CNS, microglia are programmed to respond to stimuli by upregulating transcription of a plethora of inflammatory genes, including chemokines. Microglia have also been shown to produce chemokines (*e.g.*, CCL2 and CCL5) following CNS trauma 73. These chemokines are known to direct leukocyte migration and infiltration into the injured spinal cord. We showed that PLX-treatment attenuated the injury-induced expression of CCL2, CCL3, CCL4, and CCL5 in the spinal cord. Moreover, genes that regulate blood-spinal cord barrier dysfunction (*i.e.*, MMP-12, TLR2, IL1a, etc.) were also attenuated with PLX. It is reasonable to assume that in addition to other pro-inflammatory factors that may indirectly impact leukocyte infiltration (via neuronal or vascular damage), microglial depletion directly decreased local expression of chemokines required for monocyte entry.

Although resident microglia are essential in regulating CNS development and maintenance of neuronal networks 74, studies show that long-term depletion of microglia by CSF1R inhibitors has no detrimental effects on motor activity or cognitive function 37, 42. In agreement with these reports, depletion of microglia in sham mice by PLX treatment did not lead to abnormalities in motor, cognitive, or depressive-like behavioral tasks. After SCI, PLX5622 administration did not significantly improve hindlimb locomotor function, examined using BMS scores, which is in agreement with a recent report 43 using the same treatment paradigm. Consistent with these data, histopathological analysis at 42 days post-injury showed no differences in spared whiter matter between SCI/PLX and SCI/Veh groups. SCI also caused significant dysfunction in spontaneous motor activity tested in the open field test and in motor coordination assessed by CatWalk gait analysis. Notably, PLX5622 treatment improved recovery of fine motor coordination in the mice capable of placing the plantar surface of the hindpaws, consistent with a recent report 44 in an SCI model using a different CSF1R inhibitor (GW2580).

Previous studies have investigated the effects of earlier targeting (prior to injury) of microglial responses following CNS trauma. Witcher *et al.* 75 demonstrated that depletion of microglia with PLX5622 prior to induction of central fluid percussion injury (diffuse TBI model) decreased cortical neuroinflammation at 7 days post-injury. Although it is well recognized that sustained over-activation of microglia can contribute to neurodegeneration and related neurological dysfunction 76, microglia exert beneficial effects for tissue repair especially in the early post-injury period through clearing cellular debris, producing neuroprotective factors, and directing neurorestorative processes 43. In a mouse model of stroke, depletion of microglia prior to induction of injury resulted in an increase in infarct size and dysregulated calcium responses 77. In fact, mice depleted of microglia before SCI exhibited more severe locomotor deficits (at 24 h post-injury) compared to mice treated with the vehicle diet 43, suggesting that, at least in some acute injury models, elimination of microglia that perform critical neuroprotective functions in response to CNS insult may be counterproductive. However, microglial elimination in SCI mice fed the PLX5622 diet beginning at day 3 post-injury and continuing through 5 weeks did not affect motor function 43, consistent with the present report. As moderate/severe SCI mice showed significantly decreased food consumption during the first week, the current PLX treatment paradigm resulted in only 80% and 90% microglial elimination in injured site and the brain, respectively, at 7 days post-injury as evidenced by flow cytometry analysis. While early microglial responses may be critical for neurorestoration and neuroprotection following CNS injury, partial microglia depletion in the acute stage of the injury may contribute to limited chronic neuroinflammation and improved neurological function observed in the present study.

To further delineate impact of microglial depletion on chronic SCI pathology, we performed a NanoString Neuroinflammation panel analysis of Sham or SCI mice, with or without PLX5622 treatment, to define network-level changes in the injured spinal cord at 6 weeks post-injury. Pathway analyses showed that inflammatory pathways were most significantly altered after injury.

PLX treatment affected the top 6 pathways within the theme of Immunity and Inflammation, including Inflammatory Signaling, Innate Immune Response, Microglia Function, NF-κB, Cytokine Signaling, and Adaptive Immune Response. The SCI-induced increase of chemokines, cytokines, and associated receptors was significantly reduced in the PLX treatment group. Microglia elimination in SCI mice reduced gene expression of the majority of caspase pathway (*Casp1*,* Casp4*, *Casp6*, *Casp7*, *Casp8*) as well as the pro-apoptotic gene, *Bax*, which critically impacts neuronal cell loss after SCI. In addition, a significant part of the transcriptional profile associated with astrocyte function, growth factor signaling, and matrix remodeling was also modified with PLX treatment, which may also contribute to an improved injured environment. qPCR analysis validated mRNA expression levels of microglial receptors and biomarkers of neuronal apoptosis and astrocytes. It is known that astrocyte reactivity depends on varying degrees of neuronal/axonal injury, inflammation, and vascular disruption 78. Furthermore, astrocytes and microglia communicate via cytokines and cell-surface markers during CNS injury and neurodegeneration 79. To assess the effect of microglial depletion on other glial cells, we examined GFAP, a canonical marker of astrocyte activation. The SCI-induced increase in Gfap mRNA in injured spinal cord tissue was reduced by PLX5622 treatment, suggesting reduced chronic neuroinflammation.

Across studies in human and experimental models, SCI-induced cognitive and mood disorders are consistently observed 80. In line with previous findings 13, 15, moderate/severe SCI in male mice caused significant impairment of cognitive function and depressive-like behavior. Recent preclinical work 80 has begun to elucidate the underlying mechanisms of cognitive impairments following SCI, such as neuroinflammation in the brain, distal release of a potent microglial activators (*i.e.*, CCL21) through anterograde and retrograde pathways, or systemic immune dysfunction. Post-SCI pain is also significantly correlated with neurological behavior, and can be used as a predictor of cognitive function, emotional function and quality of life 9. We and others have previously reported 14, 16, 17, 22-25 chronic inflammation in the brain after SCI in rat and mice, evidenced by predominant hypertrophic morphology of microglia, increased expression levels of pro-inflammatory cytokines and elevation of translocator protein 18 kDa - a widely used clinically as a marker of neuroinflammation 81. Our recent data 80 demonstrated that moderate/severe SCI in C57BL/6 male mice caused significantly increased levels of the proinflammatory cytokine IL6 in the brain. To determine whether there were also transcriptional changes in the brain after chronic SCI, we analyzed the cerebral cortical area using a NanoString Neuroinflammation panel analysis. We found that microglia function related genes (*i.e.*, Chn2, Chst8, Epsti1, Kcnk13, Lrrc25) were significantly increased in the cortex at 6 weeks after SCI. Although SCI did not alter the number of CD11b^+^CD45^int^ microglia in the brain at 7 days post-injury, our data indicate that SCI causes maladaptive transformation of microglia in the cerebral cortex from a neurorestorative or neuroprotective phenotype to a neurotoxic phenotype. The latter may contribute to SCI-mediated depressive-like behavior, which is in agreement with a recent study 82 showing similar changes in microglial phenotype can spur the development of neuropsychiatric diseases, including major depressive disorder. Moreover, NanoString analysis demonstrated that SCI decreased 30 out of a total of 44 differentially expressed genes in the cortex, which was strongly related to neurons and neurotransmission, apoptosis, and microglia function. Genes that showed decreased expression include a number of synaptic proteins (ionotropic and metabotropic glutamate receptors) as well as Nlgn1 and Syp, which are related to synaptic organization and synaptic vesicle function, respectively. In addition, genes related to apoptosis (anti-apoptotic gene, Bcl2) and growth factor signaling (*e.g.* Igf1) were primarily decreased in the cortex. These changes were correlated with reduced neuronal survival observed in the selected brain regions after SCI, leading to impairment of cognitive function and depressive-like behavior.

Prolonged or sustained depletion of microglia with the CSF1R inhibitors has been shown to confer neuroprotection and improve cognitive recovery in experimental models of neurological disorders including acute hippocampal lesion, Alzheimer's disease, and multiple sclerosis 38, 39, 42, 83, 84. In the present study, long-term PLX5622 treatment markedly improved spatial and working memory, as shown by improved performance in the Y maze and novel object recognition tests compared to vehicle-treated SCI mice. In addition, PLX5622-treated SCI mice showed decreased anhedonia in the sucrose preference task and less depression in the tail-suspension and forced swim tests. To determine whether dysregulated transcriptional changes in the brain after SCI could be modified by PLX treatment, the whole tissue RNA analysis in the cortical area was performed using NanoString analysis. We found that PLX treatment reduced injury-induced expression of pro-inflammatory cytokine and negative regulator of NFκB signaling, respectively. While qPCR analysis showed no significant alteration of Gfap mRNA level in the cortex by either SCI or microglial depletion, PLX5622 treatment increased the expression of a few astrocyte-related genes using a NanoString analysis. Thus, microglia depletion with PLX5622 may improve the brain microenvironment resulting in significant functional improvements following SCI. Although microglial depletion has a broad range of neuroprotective effects in neurological diseases by reducing neuroinflammation, it has also been shown to exacerbate brain neurotoxicity in mouse models of stroke, Parkinson's disease, coronavirus encephalitis 77, 85, 86. To maximize the beneficial effects of CSF1R inhibitor treatment for SCI, optimization of drug concentration and therapeutic window, as well as differences in species, need to be addressed.

However, CSF1R expression can be detected on other myeloid cells such as peripheral tissue resident macrophages, dendritic cells, neutrophils, and myeloid-derived suppressor cells. Thus, depleting cell populations other than microglia by CSF1R inhibitors might have off-target effects in various peripheral tissues including skin, adipose tissue, heart, liver, small intestine, and lung. Therefore, cautious interpretation of microglia-specific depletion effects using systemic delivery and currently available small-molecule CSF1R inhibitors is warranted. According to the National Spinal Cord Injury Database, males account for approximately 78% of new SCI cases (NSCISC, 2019). Male mice were included in the present study. However, gender-related differences after SCI have been reported with regard to neuroinflammation and neurologic functional recovery 87-90. More recently, Fukutoku *et al.*, 91 reported that female SCI mice displayed significantly more anxiety-like behaviors than those in SCI males. Microglia depletion-related sex differences in SCI-mediated brain pathology have not been reported which is intriguing for future investigation.

## Conclusion

**Figure 12:**
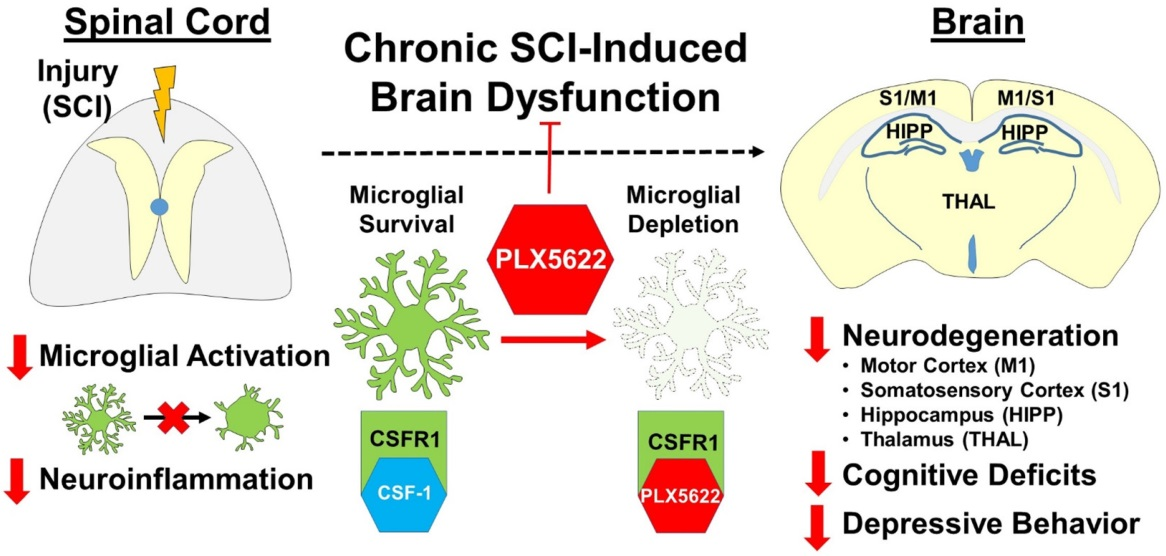
** The effect of chronic neuroinflammation on SCI-mediated brain pathology.** Delayed pharmacological microglia-deletion causes substantial changes in the spinal cord and brain transcriptomes, including those involved in neuroinflammation, leading to improved neurological recovery.

Taken together, we showed that CSF1R inhibitor PLX5622, whether initiated prior or after injury, significantly reduced inflammation in both injured spinal cord and the brain during the acute stages of injury. Chronic neuroinflammation was also reduced by PLX5622, and further supported by NanoString neuroinflammation panel analysis. These findings strongly implicate chronic neurotoxic inflammation as a major pathophysiological factor in SCI-mediated brain pathology and related neurological impairments (**Figure 12**). Furthermore, these studies support the concept that depression after SCI is not necessarily “reactive”, but rather may reflect specific neuropathological changes that can be improved by targeting neuroinflammation.

## Supplementary Material

Supplementary figures and tables.

## Abbreviations

BMS: Basso mouse scale
CSF1R: colony-stimulating factor 1 receptor
CA: cornu Ammonis
DAPI: 4',6-diamidino-2-phenylinodole
DG: dentate gyrus
DE: differentially expressed
ER: endoplasmic reticulum
FS: forced swim test
IACUC: Institutional Animal Care and Use Committee
IHC: immunohistochemistry
LFB: Luxol fast blue
NOR: Novel object recognition
OF: open field
SP: sucrose preference
SWM: spared white matter
TS: tail-suspension
PO: posterior thalamic nucleus
PLX: Plexxikon
PPI: protein-protein interaction
PCA: principal component analysis
PC1: principle component
ROS: reactive oxygen species
SCI: spinal cord injury
VPL: ventral posterolateral nucleus
VPM: ventral posteromedial nucleus
