# Supplementary material for: Delayed microglial depletion after spinal cord injury reduces chronic inflammation and neurodegeneration in the brain and improves neurological recovery in male mice
Source: Theranostics. 2020 Sep 14;10(25):11376–403. doi: 10.7150/thno.49199 (PMC7545988; doi:10.7150/thno.49199)
Supplement: Supplementary file 1 — Supplementary figures and tables. [file thnov10p11376s1.pdf]

Delayed microglial depletion after spinal cord injury reduces chronic inflammation and neurodegeneration in the brain and improves neurological recovery in male mice

Yun Li[1,*], Rodney M. Ritzel[1,*], Niaz Khan[1,*], Tuoxin Cao[1], Junyun He[1], Zhuofan Lei[1], Jessica J. Matyas[1], Boris Sabirzhanov[1], Simon Liu[1], Hui Li[1], Bogdan A. Stoica[1], David J. Loane[1], Alan I. Faden[1,2], and Junfang Wu[1,2,#]

Supplementary Material

Supplementary figures.

Supplementary Figure legends

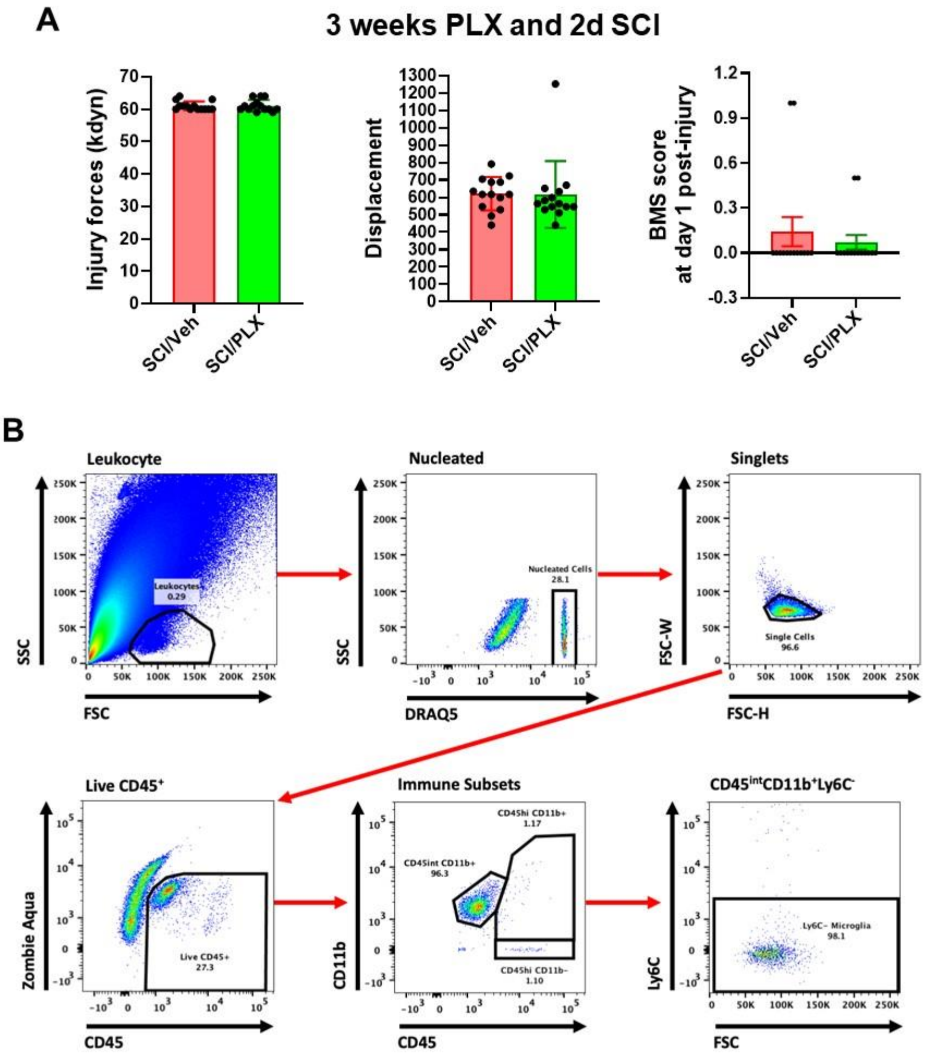

Figure S1. PLX5622 diet or vehicle chow was fed to animals three weeks before injury and remained for 2 days after injury. (A) Injury biomechanics indicated that there was no difference in injury forces, displacement, and BMS score (at 1 d post-injury) between SCI/Veh and SCI/PLX groups. N = 14 mice/group. **(B)** Representative dot plots illustrating our gating strategy for identifying $CD45^{int}CD11b^{+}Ly6C^{-}$ CNS-resident microglia and $CD45^{hi}CD11b^{+}$ infiltrating myeloid cells are shown. A splenocyte reference and Draq5 staining were used to identify single nucleated living leukocyte populations in CNS tissue.

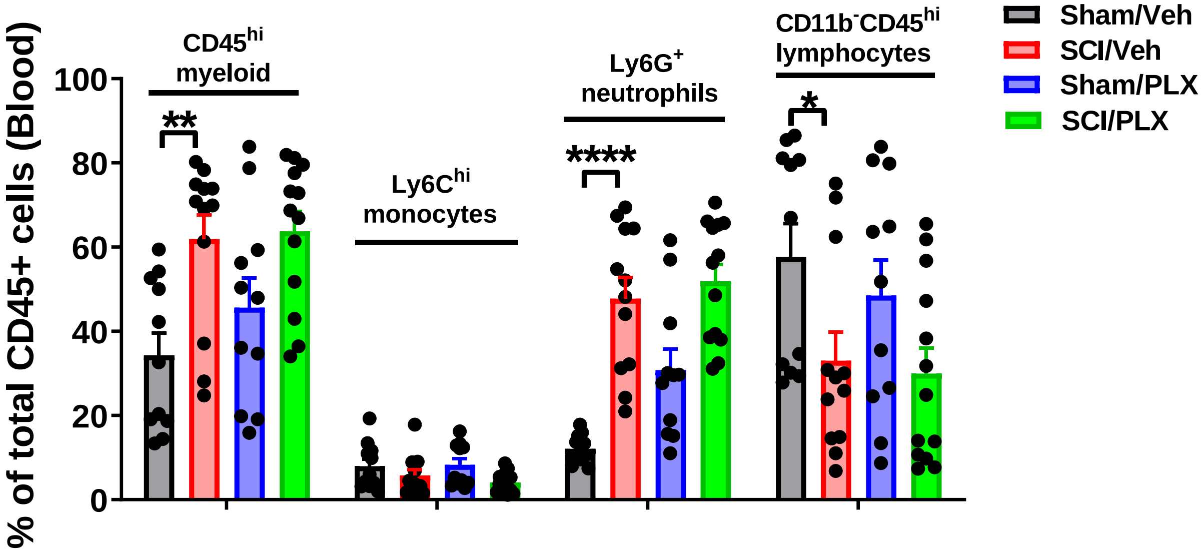

Figure S2. Systemic administration of PLX5622 did not affect circulating leukocyte populations after SCI. PLX5622 diet or vehicle chow was fed to animals beginning three weeks before injury. At 2 days post-injury, mice were euthanized and blood (200 μL) was drawn by cardiac puncture with heparinized needles. Leukocyte frequencies were quantified. SCI significantly increased circulating CDLy6C^{hi} monocytes and Ly6G^{+} neutrophils, but reduced CD11b^{-}CD45^{+} lymphocytes. However, SCI mice that had been on the PLX5622 diet (for a total of 3 weeks) did not show dysregulation in leukocyte composition. N = 11 (Sham/Veh), 12 (SCI/Veh), 11 (Sham/PLX), and 13 (SCI/PLX). 2-way ANOVA followed by Sidak's multiple comparisons test.

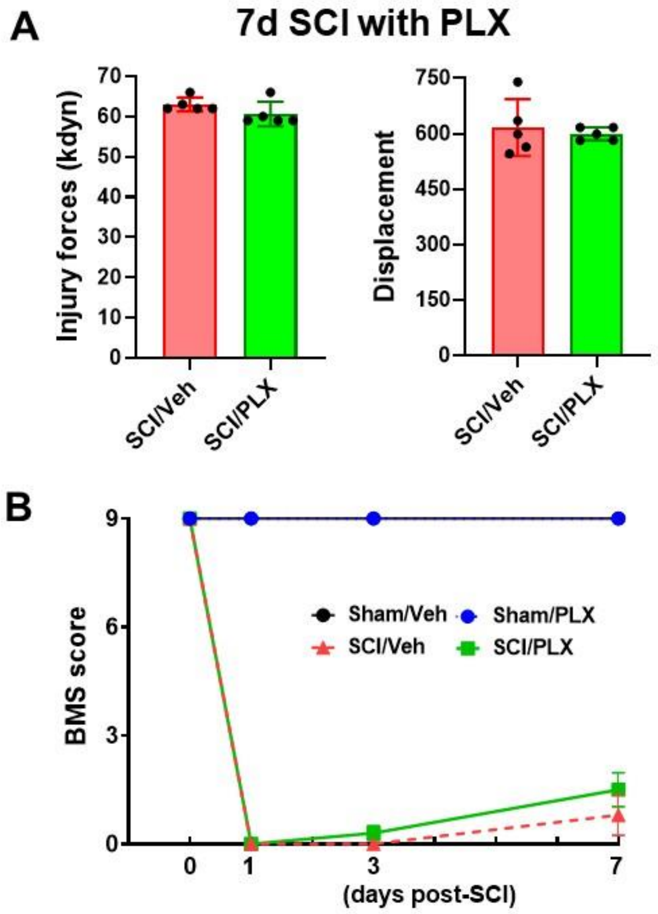

Figure S3. PLX5622 diet or vehicle chow was fed to animals on day 1 post-injury and maintained up to 7 days. (A) Injury biomechanics indicated that there was no difference in injury forces and displacement between SCI/Veh and SCI/PLX groups. N = 5 mice/group. **(B)** No significant differences were observed between two injured groups in the BMS during the first week post-injury. N = 5 mice/group.

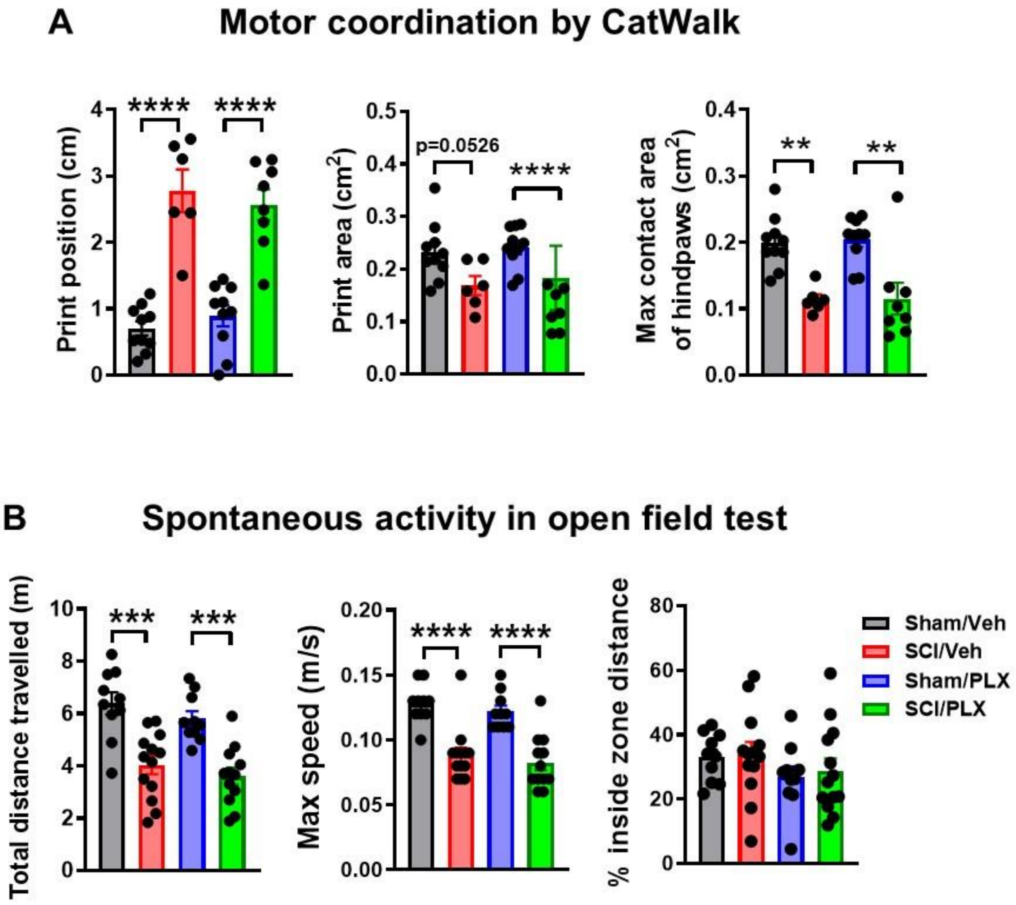

Figure S4. Gait analysis using the CatWalk apparatus and spontaneous activity in open field test. (A) Motor coordination assessed by CatWalk. All injured mice showed significant dysfunction in print position, print area and max contact area of hindpaws. There were no apparent differences in the SCI mice between PLX and Vehicle groups with regard to these parameters. PLX treatment also had no effect on motor coordination in sham mice. **(B)** Spontaneous motor activity was recorded in the open-field chamber at 5 weeks post-injury by computer-based Any-Maze automated video tracking system. SCI resulted in a significantly reduced distance traveled and walking speed, compared with sham mice. However, PLX treatment did not improve overall animal motor activity. We also did not observe the differences of percentages of inside zone distance among four groups. N = 6-10 mice/group. $^{**}p < 0.01$, $^{***}p < 0.001$, $^{****}p < 0.0001$, vs. Sham groups. 2-way ANOVA following Tukey's multiple comparisons test.

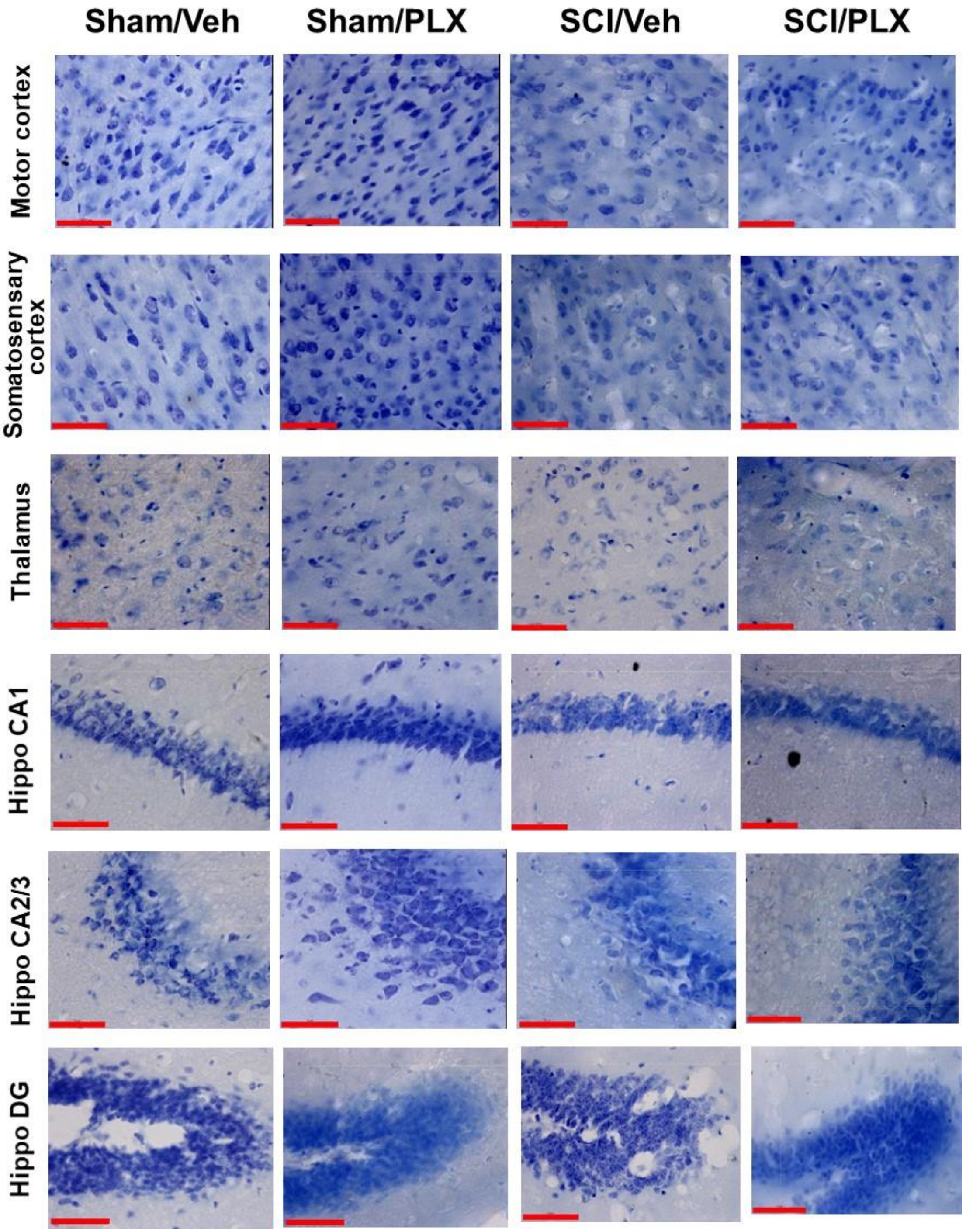

Figure S5. Representative images of cresyl violet stained neurons in the brain sub-regions including motor cortex (M1, M2), the somatosensory cortex (S1 and S2), thalamus (VPL, VPM, PO, and anterior nucleus), hippocampal CA1, CA2/3, and DG regions. Scale bar = 50 μm.

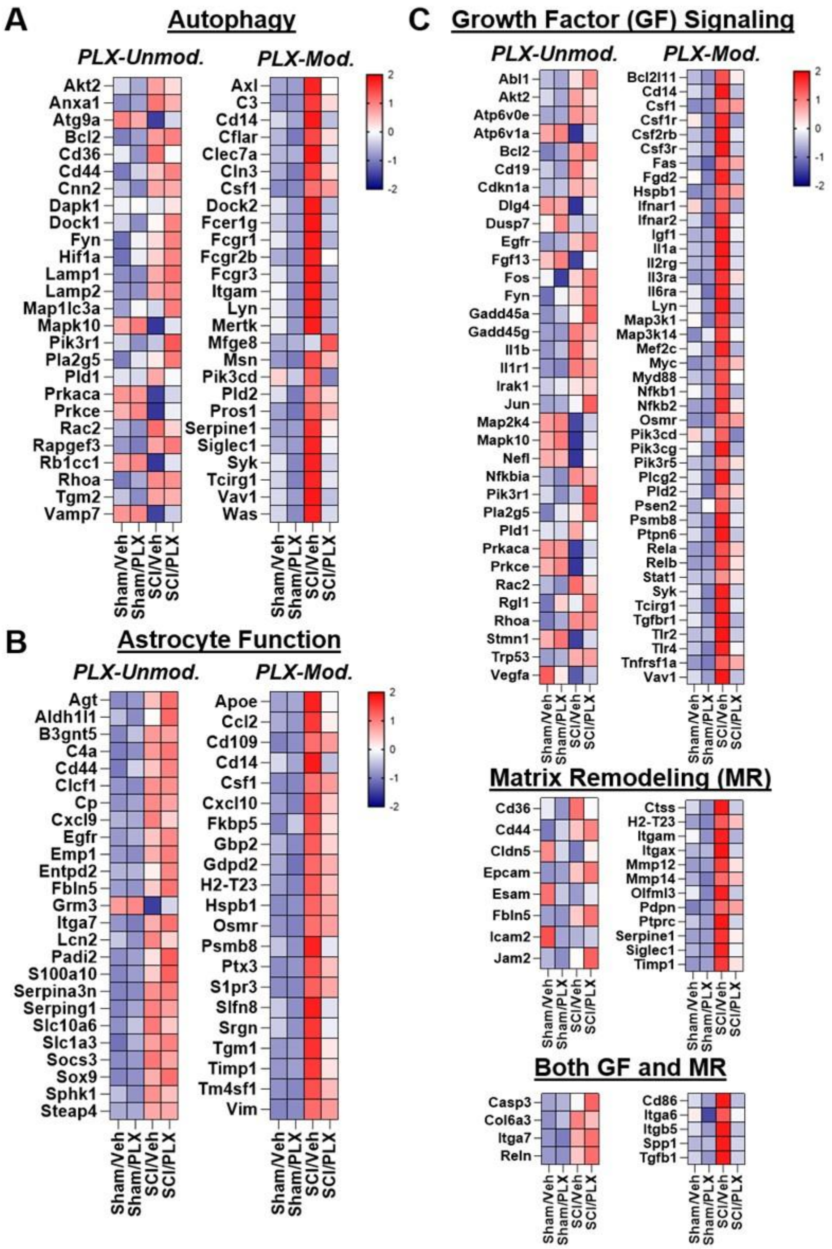

Figure S6. Long-term depletion of microglia after SCI modifies genes related to autophagy, astrocyte function, growth factor signaling and matrix remodeling. (A) Heatmap of genes related to autophagy that are differentially expressed (DE) by injury and PLX-Unmodified (left) or PLX-Modified (right). Color coding was based on z-score scaling. **(B)** Heatmap of genes related to astrocyte function that are DE by injury and PLX-Unmodified (left) or PLX-Modified (right). Color coding was based on z-score scaling. **(C)** Heatmap of genes related to growth factor signaling and matrix remodeling that are DE by injury and PLX-Unmodified (left) or PLX-Modified (right). Color coding was based on z-score scaling.

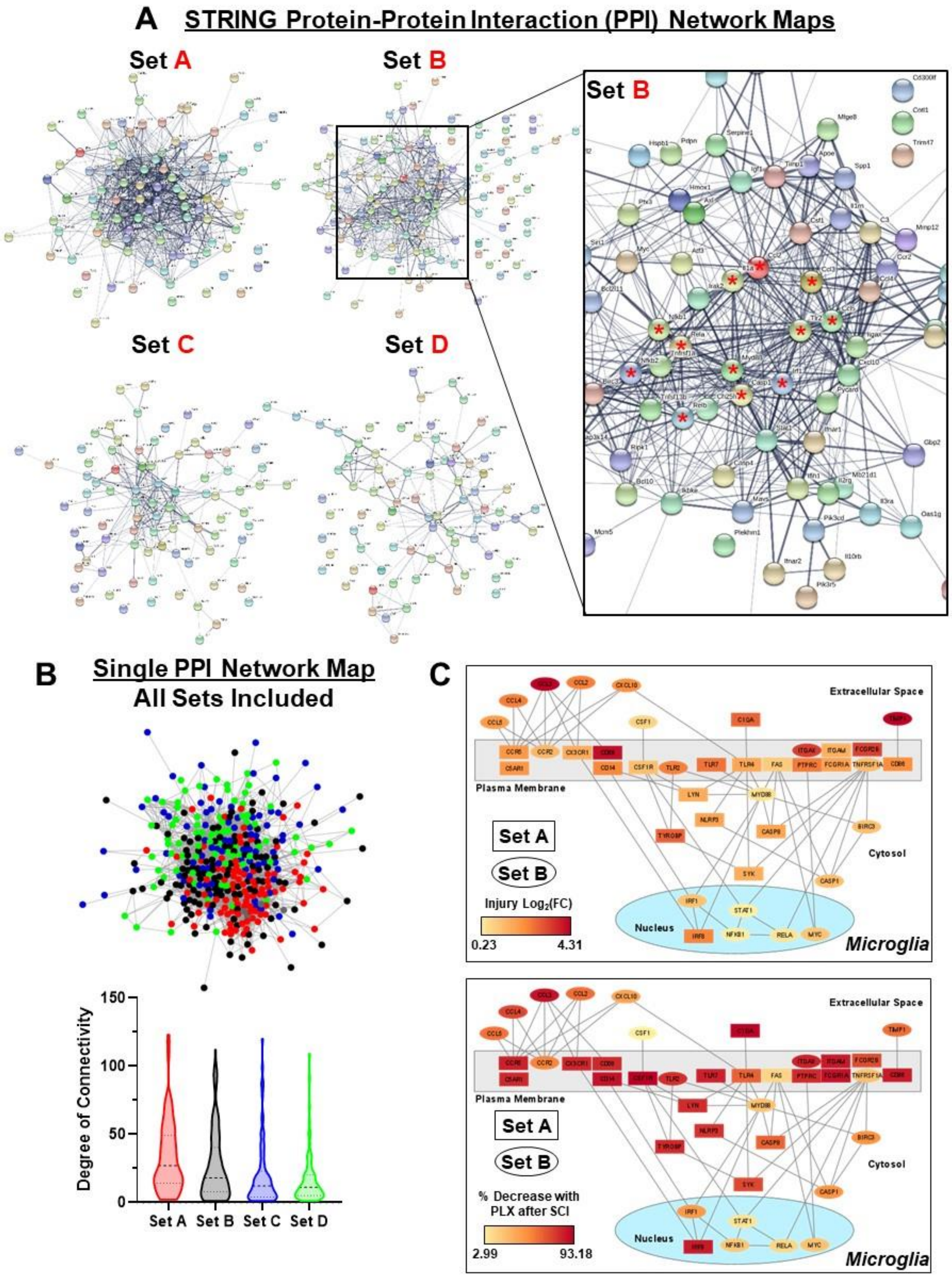
A
STRING Protein-Protein Interaction (PPI) Network Maps
Set A
Set B
Set C
Set D
B
Single PPI Network Map
All Sets Included
Degree of Connectivity
150
100
50
0
Set A
Set B
Set C
Set D
C
Extracellular Space
Plasma Membrane
Cytosol
Nucleus
Set A
Set B
Injury Log2(FC)
0.23
4.31
Microglia
% Decrease with PLX after SCI
2.99
93.18
CCL3
CCL2
CXCL10
CCL4
CCL5
CSF1
C1QA
TIMP1
CCR5
CCR2
CX3CR1
CD68
ITGAX
ITGAM
FCGR2B
C5AR1
CD14
CSF1R
TLR2
TLR7
TLR4
FAS
PTPRC
FCGR1A
TNFRSF1A
CD86
LYN
MYD88
NLRP3
CASP8
BIRC3
TYROBP
SYK
CASP1
IRF1
STAT1
IRF8
NFKB1
RELA
MYC

Figure S7. Network analysis supports the identification of inflammatory genes that are expressed in microglia specifically after SCI. (A) Protein-protein network interaction (PPI) maps for Sets A, B, C, and D generated individually from the STRING database. Genes in Set A visually demonstrated a high degree of connectivity, while Sets C and D had comparatively less. A subset of genes in Set B had a significant degree of connectivity and were identified as genes (marked with red asterisks within inset) with known biological importance in inflammatory signaling cascades after SCI including various chemokines/cytokines (*Ccl2, Ccl3, Ccl5, Il1a*), type 1 interferon response (*Irf1, Ifnar1*), inflammasome activation (*Casp1*), NFκB signaling (*Myd88, Rela/Relb, Nfkb1/Nfkb2*) and Toll-like receptor activity (*Tlr2*). **(B)** (*Top*) Single PPI map for injury genes in all Sets together and color-coded by Set membership (Set A, red circles; Set B, black circles; Set C, blue circles; Set D, green circles). (*Bottom*) The degree of connectivity, defined for a single protein as the number of direct protein interactors, witin the entire injury network was quantified for all genes in each set and presented as violin plots. Genes in Sets A and B had the greatest median degrees of connectivity (Set A = 27; Set B = 18) and higher upper ends of the distribution (Set A third quartile = 49; Set B third quartile = 40), indicating a significant amount of functional overlap between them. **(C)** Pathway interaction diagram of genes selected from Sets A (rectangles) and B (ellipses) with a degree of connectivity greater than 50 (selected based on the upper 25% of the distribution in Set A) in the entire injury PPI network map in the previous panel. The interactions between proteins that are displayed involve either direct binding or regulatory activity from Elsevier's Pathway Studio database. Interactions between various plasma membrane receptors were removed for clarity in the figure. In the top panel, protein entities are color-coded by log2(FC) with injury while in the bottom panel, protein entities are color-coded by percent reduction with PLX after SCI. These diagrams highlight the importance of chemokine, type I interferon (*Irf1, Irf8*), *Myd88-NFκB*, inflammasome (*Nlrp3, Casp1*) and Toll-like receptor signaling in the microglial response to injury.
